# Soil-Derived Inocula Enhance Methane Production and Counteract Common Process Failures During Anaerobic Digestion

**DOI:** 10.3389/fmicb.2020.572759

**Published:** 2020-10-20

**Authors:** Mira Mutschlechner, Nadine Praeg, Paul Illmer

**Affiliations:** Department of Microbiology, Universität Innsbruck, Innsbruck, Austria

**Keywords:** soil-borne methanogens, inoculation, disturbance, adaptation, process optimization

## Abstract

Although soil-borne methanogens are known to be highly diverse and adapted to extreme environments, their application as potential (anaerobic) inocula to improve anaerobic digestion has not been investigated until now. The present study aimed at evaluating if soil-derived communities can be beneficial for biogas (methane, CH_4_) production and endure unfavorable conditions commonly associated with digestion failure. Nine study sites were chosen and tested for suitability as inoculation sources to improve biogas production via *in situ* measurements (CH_4_ fluxes, physical and chemical soil properties, and abundance of methanogens) and during a series of anaerobic digestions with (a) combinations of both sterile or unsterile soil and diluted fermenter sludge, and (b) pH-, acetate-, propionate-, and ammonium-induced disturbance. Amplicon sequencing was performed to assess key microbial communities pivotal for successful biogas production. Four out of nine tested soil inocula exerted sufficient methanogenic activity and repeatedly allowed satisfactory CH_4_/biogas production even under deteriorated conditions. Remarkably, the significantly highest CH_4_ production was observed using unsterile soil combined with sterile sludge, which coincided with both a higher relative abundance of methanogens and predicted genes involved in CH_4_ metabolism in these variants. Different bacterial and archaeal community patterns depending on the soil/sludge combinations and disturbance variations were established and these patterns significantly impacted CH_4_ production. *Methanosarcina* spp. seemed to play a key role in CH_4_ formation and prevailed even under stressed conditions. Overall, the results provided evidence that soil-borne methanogens can be effective in enhancing digestion performance and stability and, thus, harbor vast potential for further exploitation.

## Introduction

Regardless of being produced in biogas reactors or natural habitats, the formation of methane (CH_4_) is driven by a delicate balance between functionally distinct microorganisms mainly from the domains of Bacteria and Archaea that are kinetically, physiologically, and thermo-dynamically linked and dependent on mutual and syntrophic interactions (Gerardi, 2003; Akuzawa et al., 2011; Schink and Stams, 2013). The anaerobic digestion (AD) process can be divided into four consecutive stages that occur simultaneously, however, they differ with regard to the resulting products and suitable conditions for the interacting microorganisms. As all four stages are microbially mediated, they are susceptible to various perturbations, with methanogenic Archaea often being considered as most vulnerable (Liu and Whitman, 2008; Traversi et al., 2011; De Vrieze et al., 2012). The main environmental factors and reactor operating conditions affecting methanogenic activity and composition include pH, temperature, volatile fatty acids (VFA), and ammonia (Chen et al., 2008; Illmer and Gstraunthaler, 2009). Hence, methanogenic communities in biogas processes are frequently maintained at mesophilic or thermophilic temperatures along with circumneutral pH values to retain process stability and prevent digester imbalance (Gerardi, 2003; Steinberg and Regan, 2008). In this context, methanogenesis has been reported to be most efficient at pH 6.5–8.2, however, the growth rate of methanogens might be substantially reduced at pH < 6.6 (Mao et al., 2015). Apart from pH, high VFA concentrations can directly or indirectly affect methanogenesis either through direct toxicity (e.g., propionate) or lowering the pH to suboptimal conditions (Gerardi, 2003; Illmer and Gstraunthaler, 2009; Franke-Whittle et al., 2014). Among VFA, propionate is probably the most toxic and its oxidation is not only energetically unfavorable (ΔG°′ of +76 kJ per mol) but also strongly affected by pH, temperature, other VFA, and reactor configuration (Gerardi, 2003; Li et al., 2012). Dogan et al. (2005), for example, reported that concentrations above 13, 15, and 3.5 g L^–1^ of acetate, butyrate, and propionate added to granular sludge led to an inhibition of methanogenesis by more than 50%, respectively. Furthermore, inhibitory concentrations of total ammonia nitrogen (TAN), which is a combination of free ammonia (FA) and its ionized counterpart ammonium (NH_4_^+^), were reported to be in the range of 1.50–7.0 g L^–1^ (Rajagopal et al., 2013). Although ammonia toxicity is a well-known problem, a “critical” concentration is seemingly difficult to define, with the discrepancies in inhibition thresholds being primarily attributed to differences in operational conditions (i.e., temperature, pH), acclimation, inocula origin, and substrate properties (Chen et al., 2008; Yenigün and Demirel, 2013; Dai et al., 2016). Regarding substrate properties, the degradation of nitrogen-rich materials, such as proteins, amino acids, and urea, can lead to high concentrations of NH_3_ and NH_4_^+^ (Chen et al., 2008), while organic wastes with a high C:N ratio may (dependent on the input material) exhibit little buffering capacity entailing the risk of a rapid pH decline, a build-up of intermediate VFA, and thus a disequilibrium in the metabolic chain (Illmer and Gstraunthaler, 2009). As input materials with a high content of fat can lead to increased production of acetate and propionate, high concentrations of VFA without an increase in alkalinity might result in adverse operational conditions and are thus associated with digestion imbalance or stress (Gerardi, 2003; Li et al., 2012). In this context, a key factor that directly influences on CH_4_ production and efficacy of the entire AD process is the choice of inoculum (Wojcieszak et al., 2017). Previous studies have shown that employing individual methanogenic organisms (e.g., *Methanosarcina* spp.) or VFA-degrading cultures helped to enhance the overall process performance of anaerobic digestion (De Vrieze et al., 2015; Mahdy et al., 2017), reduce the start-up period (Lins et al., 2014), shorten the hydraulic retention time (Baek et al., 2016), decrease the recovery period during organic overload (Tale et al., 2011, 2015), accelerate VFA degradation (Acharya et al., 2015; Town and Dumonceaux, 2016; Li et al., 2017), and increase CH_4_ production from ammonia-rich substrates (Fotidis et al., 2013, 2014). To the best of our knowledge, the application of soil-derived communities as potential inocula to improve biogas production has not been investigated until now but harbors significant benefits as they are fairly ubiquitous in nature.

Soil-borne microbial communities are exposed to a high climatic variability and hence harsh environment (Galand et al., 2003; Fischer et al., 2008), thus contradicting with the defined conditions applied in engineered methanogenic systems. Yet members of the methanogenic consortium have been detected in grasslands, forests, cold sediments of Arctic wetlands, marine sediments, acidic peatlands, and high alpine mountain areas (Peters and Conrad, 1995; Hofmann et al., 2016c, b; Praeg et al., 2017, 2019; Mutschlechner et al., 2018). Although the vast majority of methanogens are mesophilic and neutrophils (Garcia et al., 2000), several psychro-, meso-, and (hyper-) thermophilic, halophilic, and halotolerant as well as acid-tolerant species exhibiting tolerance to pH values as low as 3.8 were detected during the past years (Sizova et al., 2003; Kotsyurbenko et al., 2007; Cadillo-Quiroz et al., 2009; Bräuer et al., 2011). Hitherto more than 150 pure cultures of methanogens are described that are characterized by exceptional biochemical, metabolic, physiological, and biotechnological features enabling them to tolerate extreme environmental conditions (Taubner et al., 2015). The pervasiveness of methanogens across diverse ecosystems and, in particular, their tolerance may arise from a combination of ecological and/or community-based strategies and knowledge of these strategies might be beneficial to counteract process deterioration in anaerobic digesters without the need for cost-intensive chemical or energy inputs indispensable for process stability.

Thus, the main aims were to find out whether soil-derived communities can be used as inocula in biogas processes, and if so, whether these inocula have the potential to endure exposure to stress conditions commonly associated with digestion failure. Therefore, we investigated (i) combinations of both sterile and unsterile soil and diluted fermenter sludge (DFS) and (ii) exposure of these combinations to unfavorable conditions including (sudden) changes in pH as well as high concentrations of ammonium (NH_4_^+^), acetate, and propionate. Quantitative PCR (qPCR) targeting the gene coding for the methyl coenzyme M reductase α-subunit (*mcrA*) and amplicon sequencing targeting the hypervariable V4 region of the 16S rRNA gene were performed to resolve the contribution of key bacterial and archaeal communities steering the functioning of the overall AD process.

## Materials and Methods

### Study Sites and Sampling

Nine different study sites located in Tyrol/Austria were chosen that differed regarding their physical and chemical properties including soil pH, soil moisture, and ammonium content (Supplementary Table S1). Soil sites comprised three agricultural sites: arable land (AA), a grassland applying the liquid digestate of the biogas plant in Roppen as fertilizer (AG), and a pastureland used for grazing cattle (AP), three different forest soils: beech- (FF), larch- (FL), and spruce-dominated forest (FP), and three waterlogged sites: an alluvial soil from the river Inn (WA) as well as a raised bog (WB) and fen (low moor) (WF). Soil samples were collected in September 2017 from the top soil layer (∼10–15 cm). Three replicate plots at each study site were sampled and independently packed into plastic bags before being transported to the laboratory. Subsequently, the replicate soil samples were separately sieved (<4 mm) and stored at 4°C and −20°C for the determination of soil basic characteristics and molecular-biological purposes (qPCR), respectively as described in detail below. Concurrently with soil sampling and *in situ* CH_4_ measurements, ambient air and soil temperature were measured with a hand-held digital thermometer on site.

### Physical and Chemical Soil Properties

Dry matter content (DM) was investigated by drying 10.0 g of soil at 105°C until constant weight was obtained. Loss on ignition analysis at 550°C was used to determine the organic matter content (OM). Soil pH was determined electrochemically in a 1:2.5 soil: CaCl_2_ [0.01 M] suspension at room temperature, while the electrical conductivity (EC) was measured in a 1: 2.5 soil: deionized water suspension. The ammonium content (NH_4_^+^-N) of the soils was extracted using 2M KCl and determined colorimetrically by Berthelot’s reaction (Schinner et al., 1996). Total carbon (Total C) and nitrogen content (Total N) was evaluated on a CN analyzer (Truspec CHN Macro, Leco, MI, United States) using oven-dried soil. The determination of the soluble fractions of total carbon (TC), non-purgeable organic carbon (NPOC), and total nitrogen (TN) was performed by using a 720°C combustion catalytic oxidation method on a Shimadzu TOC-LCSH/CSN analyzer in combination with a total nitrogen measuring unit (Shimadzu TNM-L) and auto sampler (Shimadzu ASI-L) according to the manufacturer’s protocol. TC and TN were measured by diluting of the samples 1:20 with distilled water, while NPOC was determined using diluted (1:20) and acidified samples (1.5% 1M HCl) according to the manufacturer’s recommendation. TC was determined in form of CO_2_ with a non-dispersive infra-red detector, whereas TN in form of NO_*x*_ was measured using a chemiluminescence detector. Carbon hydrate-free air (Messer, Austria) with a flow rate of 150 mL min^–1^ was used as mobile phase. External standards were prepared with potassium hydrogen phthalate and ammonium chloride, respectively. All physical and chemical analyses were performed in triplicate for each sampling replicate. A summary of the determined soil characteristics is given in Supplementary Table S1.

### *In situ* Methane Measurements

Flux rates of CH_4_ were determined employing a static chamber technique according to Hofmann et al. (2016a). Three equidistant chambers (in close proximity to the soil sampling points) were inserted to a depth of 4–5 cm at each study site 10 min before *in situ* measurements were performed and were left open to the atmosphere until the sampling started. After closing the chambers with gastight butyl rubber septa, gas sampling was conducted at 0-, 15-, and 30-min intervals, with samples withdrawn from the chamber headspace with a 20 mL gas-tight syringe at each time point. After collection, the gas samples were injected into pre-evacuated Exetainer glass vials sealed with butyl rubber septa. Concentration of CH_4_ inside each vial was analyzed immediately after collection by gas chromatography on a Shimadzu GC2010 Plus (Japan) as described by Wagner et al. (2011). The *in situ* CH_4_ fluxes were scaled to a cross-sectional area of 1 m^2^ and calculated on a 30-min basis from the changes in CH_4_ concentration over time at each site, a time span during which the alteration was almost linear.

### Substrate and Inocula

Diluted fermenter sludge (DFS) derived from a plug flow digester located in Roppen, Austria served as substrate and– depending on the respective experiment– was inoculated either with an additional dose of DFS or (preincubated) soil slurries as described below. To enable liquid handling and the usage as inoculum, the sludge was diluted 1:5 with oxygen-free deionized water under anaerobic conditions prior to its use (Wagner et al., 2019a). The DFS was continuously flushed with a gas mixture of N_2_:CO_2_ (70:30) and stirred while being filled into serum flasks. When serving as substrate, the DFS was autoclaved after dilution and bottling (40 min, 121°C, 1 bar). Some physical and chemical parameters of the (undiluted) sludge derived from the inlet port of the fermenter can be found in Table 1. The methanogenic community of the DFS is dominated by *Methanoculleus* spp. and *Methanothermobacter* spp. (Illmer and Gstraunthaler, 2009; Wagner et al., 2019b). The biogas plant in Roppen treats organic wastes (green- and biowaste of 53 surrounding municipalities) with a total volume of more than 10,000 t a^–1^. A detailed description including running parameters of the fermenter can be found in Illmer and Gstraunthaler (2009).

**TABLE 1:** Physical and chemical parameters of the (undiluted) fermenter sludge (mean ± SD).

Parameter	Mean (±SD)
DM [%]	26.0 (±3.56)
OM (in DM) [%]	58.7 (±6.83)
pH	7.9 (±0.44)
NH_4_-N [mg N kg^–1^]	3200 (±460)
acetate [g kg^–1^]	1.63 (±0.90)
butyrate [g kg^–1^]	0.13 (±0.23)
propionate [g kg^–1^]	1.3 (±1.15)

*DM, dry matter; OM, organic matter; NH_4_, ammonium.*

Soil-derived inocula were prepared by filling the independently collected samples (*n* = 3) into gastight serum flasks, diluting them with sterile deionized water (1:5) and closing the flasks with butyl rubber septa (Ochs, Germany). Prior to their application as inocula, the soil slurries were pre-incubated under a headspace gas composition including N_2_:CO_2_ in a 70:30 ratio for 7 days at 37°C. Following, 5 mL of each sample was anaerobically transferred into the flasks containing 25 mL of sterilized DFS using syringe and cannula. In the case of sterile soil, the samples were treated analogously as the DFS and were autoclaved after dilution. When serving as inoculum, the diluted fermenter sludge remained unsterile and was pre-incubated in the same manner as the soil-derived inocula prior to its transfer (5 mL) into serum flasks containing 25 mL of substrate.

### Suitability of Soil-Derived Inocula

**FIGURE 1:**
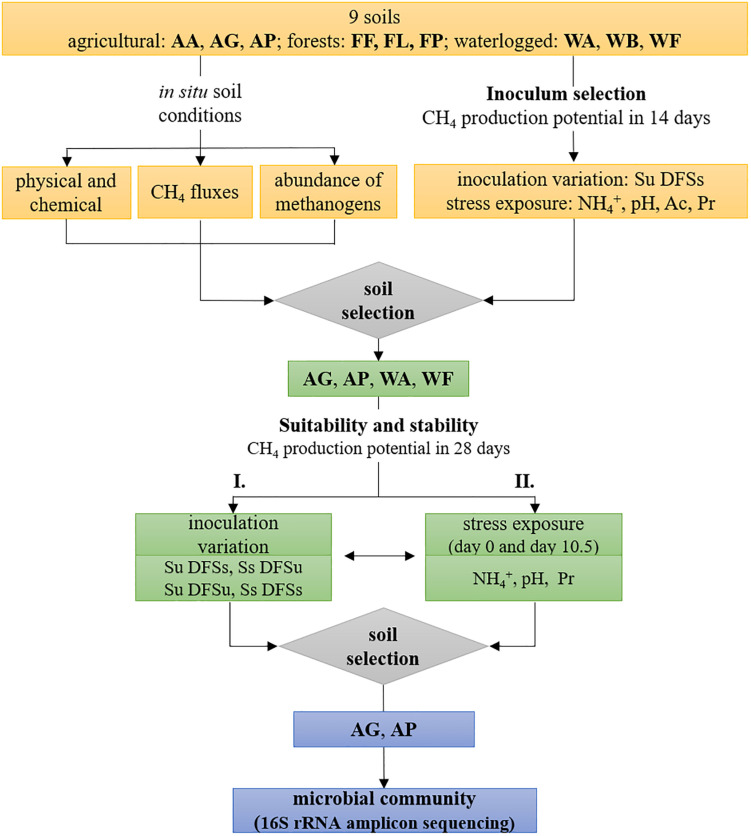
Schematic illustration of the experimental design including soil characterization, inoculum selection for anaerobic digestion using all nine initially chosen soils, and evaluation of four selected soils regarding their (i) suitability as potential inocula and (ii) stability against ammonium-, pH-, and propionate disturbance during anaerobic digestion, followed by 16S rRNA amplicon sequencing using the two most efficient soils. *AA*, arable land; *AG*, grassland with fermentation residue application; *AP*, pastureland; *FF*, beech forest; *FL*, larch forest; *FP*, spruce forest; *WA*, alluvial soil; *WB*, bog; *WF*, fen; *Ac*, acetate; *Pr*, propionate; *Su*, soil unsterile; *Ss*, soil sterile; *DFSu*, diluted fermenter sludge unsterile; *DFSs*, diluted fermenter sludge sterile.

The experimental setup is schematically illustrated in Figure 1 and was divided into two major tasks, with the first task mainly dealing with the evaluation if (anaerobic) soil-derived communities are suitable inocula for AD processes. During a preliminary screening (inoculum selection), all nine initially chosen sites were tested during the first incubation (Figure 1). The suspended and pre-incubated soil samples (5 mL each) were mixed with 25 mL autoclaved and diluted fermenter sludge serving as substrate (Soil unsterile and Diluted Fermenter Sludge sterile, Su DFSs) as described previously. The soil inocula were prepared with the three independently sampled replicate plots of each study site in three technical parallels (*n* = 9 per site). Following, the samples were anaerobically incubated at 37°C for 14 days, with gas samples being withdrawn on day 0, 3.5, 7, and 14 to determine the gas composition in the headspace.

In the next step, four soils were selected upon methane production efficiency and used in another test series consisting of four different treatments, which included variations of soil and/or DFS inocula as follows: soil unsterile and DFS sterile (Su DFSs), soil and DFS unsterile (Su DFSu), soil sterile and DFS unsterile (Ss DFSu), and soil and DFS sterile (Ss DFSs) as shown in Figure 1. Unsterile soil and/or DFS served as inocula, while sterile DFS served as substrate. To verify their sterility, DFS and soil samples were autoclaved and incubated individually under the same conditions as the samples combining soil and DFS and repeatedly analyzed for methane production. GC measurements were performed on day 0, 3.5, 7, 10.5, 14, and 28. Additional liquid samples were taken on day 28 and stored frozen until used for amplicon sequencing using two of the four soils selected upon CH_4_ production dynamics (Figure 1).

### Stability Against Common Stress Factors

After evaluating the suitability of soil-derived inocula, common stress factors, i.e., NH_4_^+^-content, initial pH, and VFA concentrations were varied using all nine initially chosen soils as inoculum to find out whether they have the potential to counteract process disturbance (Figure 1). Therefore, the mixed soil and DFS samples (Su DFSs) were amended with 5 mL of sterile anoxic stock solutions to reach a final concentration of 200- and 400 mM L^–1^ acetate or 170- and 340 mM L^–1^ propionate (both added as sodium salt), as well as 2 and 4 g L^–1^ NH_4_^+^-N (added as NH_4_Cl to minimize pH effects), respectively. The concentrations used in the present study were based on a 2-year investigation in the same biogas plant (Illmer and Gstraunthaler, 2009), with the lower ones representing the concentration where an inhibition started to take place (the higher ones represent twice the lower concentrations). Considering their potential inhibitory effect, we wanted to test if the involved organisms are adapted/or tolerant to high VFA concentrations and whether changes in the community structure occur, e.g., shifts in the prevalent acetoclastic pathway as *Methanosarcina* spp. are considered predominant at higher acetate concentrations (De Vrieze et al., 2012). The stock solutions were prepared in sterile serum flasks containing anoxic deionized water (boiled and flushed with N_2_) and added by using syringe and cannula. The pH variants were established by adjusting the mixed and diluted samples to an initial pH of 5.0 and 6.0 using 1.0 M HCl, respectively (*n* = 9). Samples without any further addition served as controls (pH soil and DFS combined: 7.19) and were amended with 5 mL of sterile, deionized water to reach an equivalent volume in all flasks.

Subsequently, another test series using the four previously selected soil inocula in different combinations with DFS (Su DFSs, Su DFSu, Ss DFSu) was performed to evaluate the adaptive capability of the indigenous communities to high NH_4_^+^ and propionate concentrations (4 g L^–1^ and 340 mM L^–1^, respectively) as well as a suboptimal pH of 6.0 (adjusted with 1.0 M HCl) during start-up (*n* = 3) (Figure 1). Therefore, anoxic stock solutions were prepared as described previously and added to the soil slurries using syringe and cannula. Samples without any inhibiting additive again served as controls (initial pH 7.19) that were treated equally as stated previously. The sterile variants of the respective samples were again established by autoclaving. Gas samples were withdrawn on day 0, 3.5, 7, 10.5, 14, and 28 (end of anaerobic incubation) and analyzed via GC, the pH was measured concurrently with sampling. In addition, an aliquot of the liquid samples was taken on day 0 (VFA analyses) and at the end of anaerobic incubation (VFA and amplicon sequencing) and stored frozen until further processing.

For evaluating the response to stress at different time points, another experiment was conducted using the four chosen soils as respective inoculum (Su DFSs), which were stressed either with ammonium (4 g L^–1^), propionate (340 mM L^–1^), or a pH drop (6.0). Each stress factor was added either on day 0 (variant A) or on day 10.5 (variant B). Irrespective of the starting point of stress exposure, the samples were incubated for 28 days and measured on day 0, 3.5, 7, 10.5, 14, and 28. Regarding pH variation, 1.0 M HCl was used to decrease the pH to 6.0 on day 0 (variant A) or day 10.5 (variant B). Control samples without any further addition (initial pH 7.19) and anoxic stock solutions were prepared analogously as described above, with the latter again being added using syringe and cannula at the two different starting points.

### Analytical Methods

The gas composition was evaluated using a Shimadzu GC2010 gas chromatograph (Japan) equipped with a flame ionization detector (CH_4_) and thermal conductivity detector (CO_2_, H_2_) as described in Wagner et al. (2011). The volume of the produced gas was calculated based on the overpressure in the flasks (measured with a digital precision manometer; GDH 200-13 Greisinger electronic, Germany) and ambient pressure [data derived from Zentralanstalt für Meteorologie und Geodynamik (ZAMG), Austria]. The pH of the slurries during anaerobic incubation was measured with pH indicator stripes (Macherey-Nagel).

### DNA Extraction and *mcrA*-Targeted Quantitative PCR (qPCR)

Genomic DNA was extracted from 0.10 g from three replicates of soil samples prior to incubation (*in situ* samples) using the NucleoSpin^®^ Soil Kit (Macherey-Nagel) according to the manufacturer’s instructions. Subsequently, the quantity and purity of the extracted DNA were evaluated via UV/VIS spectrophotometry with NanoDrop 2000cTM (PeqLab, Germany) and QuantiFluor^®^ dsDNA Dye (Promega, Germany).

Quantitative PCR (qPCR) was conducted on a Corbett Life Science (Qiagen, Netherlands) Rotor-Gene Q system using the SensiMix SYBR No-Rox Kit (Bioline, United Kingdom). qPCR of methanogenic Archaea targeted the gene coding for the methyl coenzyme M reductase α-subunit (*mcrA*), with the primers mlas and *mcrA*-rev being applied (Steinberg and Regan, 2008). *Methanosarcina thermophila* (DSM 1825, Steinberg and Regan, 2008) served as standard. The qPCR mix contained (per 20 μL): 5.28 μL PCR grade water, 10.00 μL SensiFAST^TM^ Probe No-ROX (Bioline, United Kingdom), 0.40 μL MgCl_2_ (50 mM), 0.38 μL of each primer (10 μM), 0.8 μL enhancer (5x), and 2 μL template (diluted to 2.0 ng μL^–1^). Prior to amplification, the samples were subjected to an initial denaturation step at 95°C for 10 min, followed by 45 cycles of 30 s at 95°C, 30 s at 66°C, and 30 s at 72°C. Each run included negative controls (*Escherichia coli*) and non-template controls (UltraPure DNase/RNase-Free Distilled Water, Invitrogen, United States). After quantification, PCR products were checked via melting curve analysis. Concentrations of the standard DNA were determined by using the Quant-iT PicoGreen ds-DNA reagent (Invitrogen, United States) according to the manufacturer’s instructions. *mcrA* gene copy numbers were calculated as stated by Yu et al. (2005).

### Sequencing Library Preparation and 16S rRNA Amplicon Sequencing

DNA was extracted from the enrichments combining Su DFSs, Su DFSu, and Ss DFSu after 28 days of anaerobic incubation and from pre-incubated and diluted soil and diluted fermenter sludge in triplicate. Therefore, the samples were centrifuged for 10 min at 20,000 × *g*, with the NucleoSpin^®^ 96 soil kit and NucleoVac 96 Vacuum Manifold (Macherey-Nagel) being subsequently used according to the manufacturer’s protocol. After extraction, the quality and purity of the extracted DNA were checked via UV/VIS spectrophotometry with NanoDrop 2000cTM (PeqLab, Germany). The universal primer pair 515f/806r (Apprill et al., 2015; Parada et al., 2016) targeting the V4 region of the 16S rRNA gene was used for constructing PCR amplicon libraries as described in Prem et al. (2019). To validate the performance of the entire library construction and sequencing process, a mock community (ZymoBIOMICS Microbial Community Standard, Zymo Research, United States) was included in all steps until final data processing. Library preparation comprised two consecutive PCR runs. During PCR I, the primer pair 515f/806r was used for adapter ligation by conducting an initial denaturation phase of 30 s at 98°C (heat activation of the proof-read polymerase), followed by 30 cycles of 10 s at 98°C, 30 s at 58°C, 20 s at 72°C, and a final step at 72°C for 2 min in a Thermocycler T100 (Bio-Rad Laboratories, United States). The PCR I mix contained per 25 μL reaction volume: 12.5 μL NEBNext^®^ Mastermix (New England Biolabs, United States), 1.25 μL of each primer [final concentration 0.5 μM], and 10 μL template (diluted to 0.5 ng dsDNA μL^–1^). Template DNA was quantified using a Quant-iT^TM^ PicoGreen^TM^ dsDNA Assay Kit (Invitrogen, Waltham, MA, United States) and the multimode fluorometer Zenyth3100 (Anthos, Salzburg, Austria). During PCR II, the barcoded library was prepared with an initial denaturation step of 30 s at 98°C and running 5 cycles of 10 s at 98°C, 30 s at 57°C, 20 s at 72°C, and 2 min at 72°C. The PCR II mix contained per 20 μL reaction volume: 10 μL NEBNext Ultra II Q5 Master Mix, 1 + 1 μL of the respective barcode primer pair (final concentration 0.5 μM), 3 μL PCR grade water, and 5 μL of 1:5 diluted PCR I products. The success of both PCR runs was checked via agarose gel electrophoresis (1.5% agarose, 15 min, 100 V) and PCR products were quantified using a Quant-iT PicoGreen dsDNA Assay Kit (Invitrogen, Waltham, MA, United States) and a multimode fluorometer Zenyth3100 (Anthos, Salzburg, Austria). Subsequently, the PCR products were pooled and the library prepared with equal amounts of DNA per sample with a concentration of 47 ng μL^–1^. PCR products were purified using the HiYield^®^ Gel/PCR DNA Fragment Extraction Kit (SLG, Germany). Finally, 100 μL of the library were sent to Microsynth AG (Balgach, Switzerland) for amplicon sequencing (Illumina MiSeq 2 × 250 bp paired end read). During pre-processing of the raw data, forward and reverse reads were combined (aligned) by using the make.contigs command in MOTHUR (Schloss et al., 2009). During the make.contigs command, sequences of the forward and reverse primers are provided as well and were, thus, removed. Stringent filtering steps were performed to remove sequences with ambiguous reads (>6 homopolymers and a length of <240 bp or >275 bp). After quality filtering, unique sequences were aligned against the SILVA rRNA gene database (release 132) (Quast et al., 2012), chimeric amplicons were removed applying Uchime (Edgar et al., 2011). Sequence classification was carried out using the Wang approach (Wang et al., 2007). Operational taxonomic units (OTUs) were binned at 97% identity using the OptiClust algorithm introduced in MOTHUR (Schloss et al., 2009). The sequence counts were subsampled to the smallest sample size of 11,680 reads per sample. Bioinformatics were performed using MOTHUR v.1.39.0 (64 bit executable) (Schloss et al., 2009). Diversity and richness indices were calculated using MOTHUR (Schloss et al., 2009), with Shannon diversity index and Chao I richness (α diversity) being estimated based on OTU abundance matrices rarefied to the lowest number of sequences. Biomarker discovery was performed using the LEfSe (Linear discriminant analysis effect size) command in MOTHUR to reveal significant differences in the archaeal and bacterial communities between the soil/DFS variants and stress factors. LEfSe is an algorithm for high-dimensional biomarker discovery and explanation that identifies taxa characterizing the differences between two or more biological conditions (or classes) (Segata et al., 2011). OTUs with a linear discriminant analysis (LDA) log score > 3.7 were considered for interpretation. Significant genera identified for control and stress variants were analyzed and visualized with STAMP 2.1.3 (Parks et al., 2014) using White’s non-parametric *t*-test (two-sided) to distinguish between variants (White et al., 2009). The confidence intervals were provided via percentile bootstrapping (1000 permutations) and the false discovery rate was controlled by using the *Benjamini–Hochberg* procedure (B-H adjustment) (Benjamini and Hochberg, 1995).

All sequence data obtained in this study were submitted to the National Center for Biotechnology Information (NCBI) Sequence Read Archive (SRA) and are accessible from the NCBI repository under BioProject PRJNA637206.

### PICRUSt Analysis for Predicted Metabolic Functions

For functional predictions, metagenome functional content was predicted from 16S rRNA genes by using the software package PICRUSt (phylogenetic investigation of communities by reconstruction of unobserved states) as described in Langille et al. (2013). Therefore, OTUs were picked searching against the Greengenes reference (Greengenes v13.5) (DeSantis et al., 2006). After picking OTUs for the use in PICRUSt, the OTU table was normalized by copy number and the metagenome predicted on the output of the normalized OTU table by using the script normalize_by_copy_number.py and predict_metagenomes.py, respectively, on the Galaxy server of the Huttenhower Lab (v1.0.0). Metagenomes were predicted from the PICRUSt-formatted, characterized-protein functional database of KEGG (Kyoto Encyclopedia of Genes and Genomes) Orthology. The predicted functions were classified as KEGG Orthologs (KOs) resulting in 6,909 KOs across all samples. The KOs were further categorized by function on a KEGG Pathway Hierarchy Level of 3.

### Statistical Data Treatment

Statistical analysis was performed by using the software package Statistica 12.0 (StatSoft^®^), SigmaPlot 14.0 (Systat Software Inc.), PAST^®^ 3 (Hammer et al., 2001), and Microsoft Excel^®^. Unless otherwise specified, results are given as mean ± standard deviation, *n* = 3 or *n* = 9. Significant differences were observed by one-way or multifactorial ANOVA. A significance level of 0.05 was used to assess differences between treatments. The Bonferroni test was used to discriminate between single variants. Kruskall–Wallis ANOVA and Mann–Whitney *U* test were performed in case of non-normally distributed data. Spearman correlation analyses (Spearman rs) of VFA, CH_4_, and sequencing data were performed at genus level: volatile fatty acid and CH_4_ data were log (x + 1), and the OTU data box-cox (x + 1) transformed.

## Results

### Site Characteristics and Soil Physical and Chemical Parameters

While the agricultural and forested sites exhibited a significantly higher dry matter content than the waterlogged sites, the latter were characterized by significantly higher organic matter as well as total C- and N-contents (Supplementary Table S1). Agricultural and waterlogged sites both shared similar values regarding electrical conductivity, whereas the forest soils showed significantly lower values. TC and NPOC turned out to be lowest in the agricultural sites but these sites shared similar values with the forest sites regarding TN. Acidic to slightly acidic pH values were detected in the forest sites (ranging from pH 3.87 to pH 6.64). All other soils had pH values in the (near) neutral range. The NH_4_^+^ content turned out to be similar in all studied sites, with no significant differences being detected across the different soil types (Supplementary Table S1).

### Part I: Selection of Soils as Suitable Inocula

**FIGURE 2:**
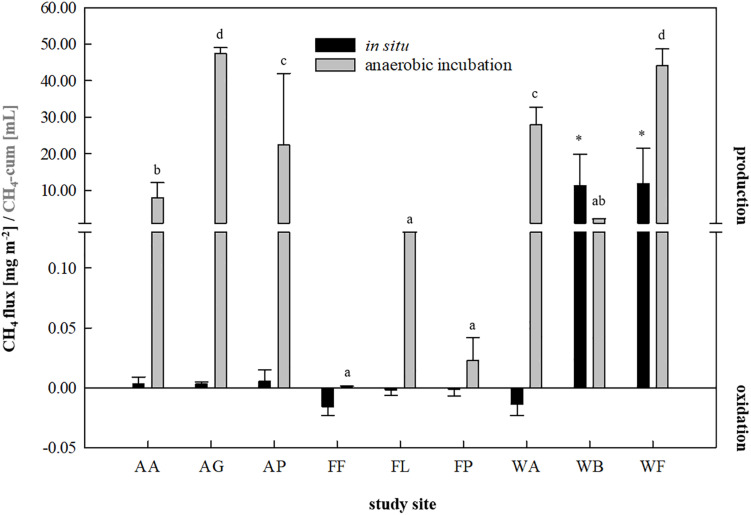
*In situ* methane fluxes [mg CH_4_-C m^–2^, *n* = 3] in 30 min and cumulative methane production [mL, *n* = 9] in 14 days of anaerobic incubation of each study site. Agricultural sites: *AA*, arable land; *AG*, grassland; *AP*, pastureland. Forest sites: *FF*, beech; *FL*, larch; *FP*, spruce. Waterlogged sites: *WA*, alluvial soil; *WB*, bog; *WF*, fen. Significant differences (*p* < 0.05) in cumulative CH_4_ production dependent on soil inocula are indicated by different characters, asterisk indicate significantly higher *in situ* CH_4_ fluxes.

For inoculum selection, all nine soils were first investigated for their ability to produce CH_4_ *in situ* as well as during a fortnightly anaerobic incubation using the soils as inocula and sterile DFS as substrate (Figure 1). A summary of the calculated fluxes is presented in Figure 2. Although the agricultural sites were characterized by significantly higher dry matter contents compared with waterlogged sites (Supplementary Table S1), CH_4_ production turned out to be favored over CH_4_ oxidation (with weak but positive fluxes ranging from +0.0031 to +0.0051 mg CH_4_-C m^–2^ on average) (Figure 2). The studied fen (WF) and bog (WB) both showed highly positive CH_4_ fluxes during 30 min of measurement (+11.86 and +11.30 mg CH_4_-C m^–2^ on average, respectively), while the studied alluvial soil (WA) was characterized by CH_4_ consumption (−0.014 mg CH_4_-C m^–2^ on average). Similarly, all three forest sites revealed negative CH_4_ fluxes that ranged from −0.002 to −0.014 mg CH_4_-C m^–2^ on average (Figure 2). In this context, the CH_4_ sink strength in the forest soils decreased according to the following sequence: beech (FF) > larch (FL) > spruce (FP).

Regarding anaerobic incubation of the samples combining Su and DFSs without disturbance (Figure 2), all three forest soils as well as the samples derived from the arable land (AA) showed very low to negligible CH_4_ production potential and were thus not considered as potential inocula for AD processes. In contrast to *in situ* quantification revealing highly positive CH_4_ fluxes, the studied bog (WB) also showed low CH_4_ production potential during anaerobic enrichment reaching 2.14 ± 0.04 mL in 14 days under non-disturbed conditions and was therefore excluded from further investigation. Two of the agricultural (AG: 47.39 ± 1.62 mL and AP: 22.43 ± 17.75 mL on average) and two waterlogged sites (WA: 27.96 ± 6.01 mL and WF: 44.17 ± 4.61 mL on average), however, revealed significantly higher methane production potentials during 14 days of anaerobic incubation compared with the other sites (Figure 2) and were thus considered as suitable soil-derived inocula.

**FIGURE 3:**
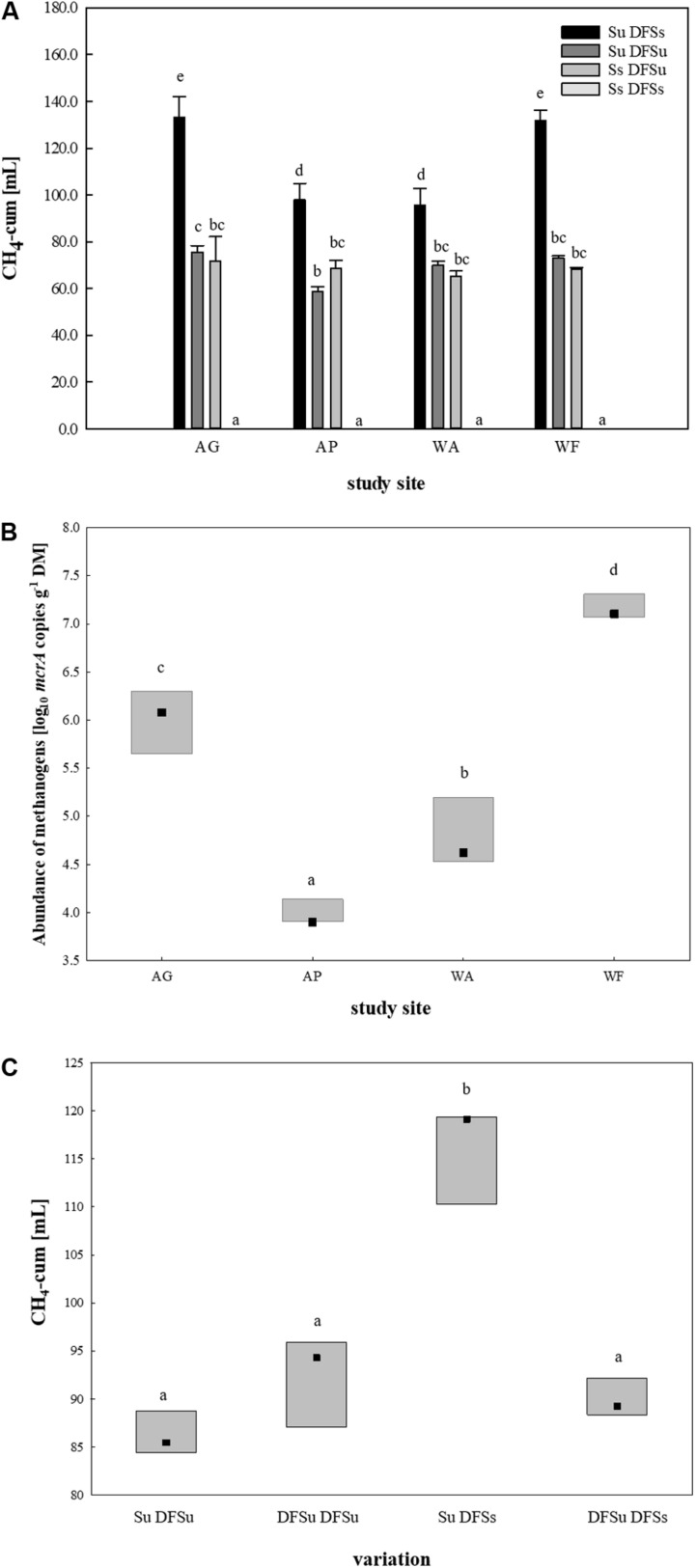
**(A)** Cumulative CH_4_ production [mL] in 28 days of anaerobic incubation (undisturbed) in four soils using different inoculations. Results are given as mean (±SD), *n* = 3. **(B)** *mcrA* targeted qPCR in the four selected soils. Box: median with data range (minimum to maximum), *n* = 3. Significant differences (*p* < 0.05) are indicated by different characters. *AG*, agricultural grassland; *AP*, agricultural pastureland; *WA*, waterlogged alluvial soil; *WF*, waterlogged fen. *Su*, soil unsterile; *Ss*, soil sterile; *DFSu*, diluted fermenter sludge unsterile, *DFSs*, diluted fermenter sludge sterile; *DM*, dry matter. **(C)** Cumulative CH_4_ production [mL] during 28 days of anaerobic incubation dependent on soil/DFS variation using AG as soil-derived inoculum. Box: median with data range (minimum to maximum), *n* = 3. Significant differences (*p* < 0.05) are indicated by different characters. *Su*, soil unsterile; *Ss*, soil sterile; *DFSu*, diluted fermenter sludge unsterile; *DFSs*, diluted fermenter sludge sterile.

After restricting to four soils, suitable inocula were tested for their potential in enhancing biogas production by using combinations of either sterile and unsterile soil and DFS (Su DFSs, Su DFSu, Ss DFSu, and Ss DFSs). These combinations were anaerobically incubated for 4 weeks without disturbance (Figure 1), the obtained cumulative CH_4_ production during 28 days is shown in Figure 3A. Remarkably, a significantly higher CH_4_ production (∼40–50%) could consistently be achieved in the samples using unsterile soil as inoculum combined with sterile DFS as substrate (Su DFSs) compared with the variants using unsterile DFS, which applied for all four tested soil inocula, i.e., soil inoculation appeared to be even more effective in improving CH_4_ production than (bioaugmented) DFS. Irrespective of the tested soil inoculum, no methane was produced during the entire incubation period when sterile soil was combined with sterile DFS (Figure 3A). Interestingly, the samples using AG and WF as well as AP and WA as inocula shared similar CH_4_ production patterns during 28 days of anaerobic incubation (Supplementary Figure S1).

Following, *mcrA* targeted qPCR was conducted using native soil samples of the four chosen study sites (Figure 3B). Consistent with the cumulative CH_4_ production rates, the grassland fertilized with liquid digestate from the reactor revealed a significantly higher abundance of methanogenic Archaea than the pastureland (LOG_10_ abundances of 6.0 ± 0.29 and 4.0 ± 0.12 gene copies g^–1^ DM, respectively). The same applied for the two water-saturated soils, with the alluvial soil (WA) revealing a significant lower LOG_10_ abundance of 4.8 ± 0.31 gene copies g^–1^ DM compared with the fen with a LOG_10_ abundance of 7.2 ± 0.11 gene copies g^–1^ DM on average (Figure 3B).

**FIGURE 4:**
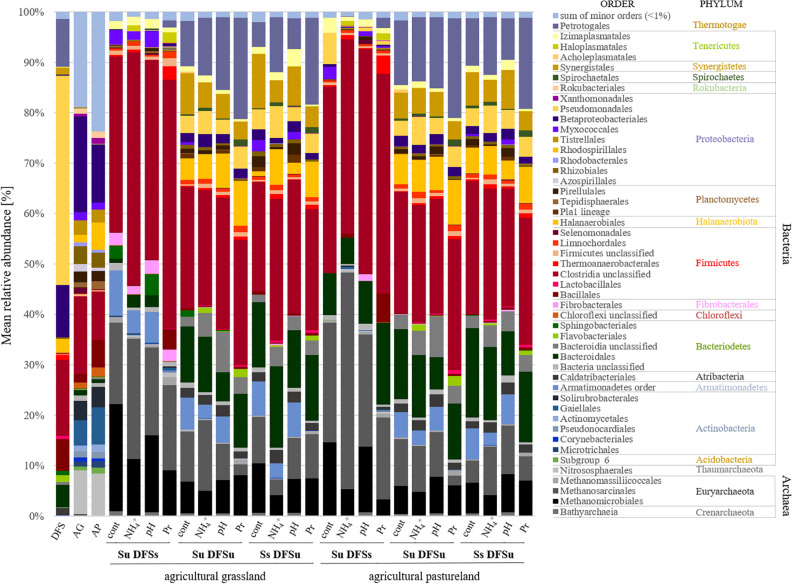
Mean relative abundance [%] of prokaryotic communities at order (and phylum) level depending on different inoculations and stress variations (*n* = 3). *DFS*, diluted fermenter sludge; *AG*, agricultural grassland; *AP*, agricultural pastureland; *cont*, controls; *Pr*, propionate; *Su*, soil unsterile; *Ss*, soil sterile; *DFSu*, diluted fermenter sludge unsterile, *DFSs*, diluted fermenter sludge sterile.

Due to the significantly higher cumulative CH_4_ production in the samples consisting of unsterile soil and sterile DFS, we wanted to test if and to what extent a disintegration of the substrate by autoclaving is responsible for the observed improvement. Therefore, a further set of experiments was performed using the agricultural grassland (AG) as inoculum and combining sterile and/or unsterile soil and DFS, which resulted in the following combinations in triplicate: Su DFSu, Su DFSs, DFSu DFSu, and DFSu DFSs (inoculation with unsterile DFS). Once again, the highest cumulative CH_4_ production of 116.24 ± 5.17 mL in 28 days was obtained in the samples combining unsterile soil and sterile DFS (Figure 3C), which corresponds to an increase of about 30% compared with the other combinations that again showed quantitatively similar CH_4_ production potentials (Figure 3C). Taxonomic identification of the prokaryotic community composition in both soil (AG and AP) and DFS samples at phylum level indicated that Proteobacteria (∼53%; mainly γ-Proteobacteria) and Firmicutes (∼23.5%; mainly Clostridia) accounted for the largest proportion in the DFS samples *per se*, followed by Thermotogae (∼9.6%) that were absent in the two soil samples AG and AP (Figure 4). The same applied for Synergistetes that, however, had a relatively small share (∼2%) in the total bacterial community. Similar to the DFS samples, Proteobacteria (34.1 and 30.5%, respectively) and Firmicutes (22.4 and 15.6%, respectively) substantially contributed to the bacterial communities of the two soils, however, also Actinobacteria ranked with a share of about 20% among the most abundant bacterial phyla (Figure 4). Regarding the total archaeal composition, Methanomicrobiales were predominant in DFS samples, while Methanosarcinales were predominant in soil samples, however, they were only detected in low abundance (<1%) in the samples prior to anaerobic incubation (and thus summarized as rare). The low abundance of methanogens, particularly in the DFS samples, is most probably the result of sampling, storage and preparation of the sludge during which oxygen exposure cannot entirely be prevented. Beyond, the DFS samples derived out of material from the input of the fermenter and were used for amplicon sequencing prior to anaerobic incubation (to allow equal conditions to the soil samples), however, methanogenic organisms could be readily activated under favorable conditions. Aside from Euryarchaeota, the typical soil-associated phylum Thaumarchaeota (primarily Nitrosphaerales associated with ammonia oxidation) accounted– with a substantial share of 8.5% (AG) and 8.1% (AP) on average– for a major proportion of the soil archaeal community, next to Methanocellales that were only present in both soils (emphasizing their adaptation to aerated environments). In this context, estimated Shannon diversity and Chao I richness (α-diversity) indices for all approaches revealed that the lowest diversity and richness could be detected in the DFS samples (Chao I: 1267.79 ± 291.60, Shannon: 3.29 ± 0.26), whereas the highest community diversity was observed in the two soil samples (Chao I: AG 5669.25 ± 1134.24 and AP 5467.07 ± 439.64, Shannon: AG 6.04 ± 0.34 and AP 6.24 ± 0.12) prior to incubation, which could indicate a high number of rare (but highly specialized) species (data not shown).

The dominant orders (average relative abundance ≥ 1%) responsible for the characteristic differences in the variation-associated prokaryotic community patterns are summarized in Figure 4. Among Bacteria, the most distinct structural differences between the samples using unsterile and sterile soil as inocula at order level related to Clostridiales (Firmicutes), which were significantly more abundant in the samples using unsterile soil. Contrarily, Petrotogales (Thermotogae) and Synergistales (Synergistetes) were indicative for the samples combining sterile soil with unsterile DFS and were among the most significant LEfSe-detected bacterial biomarkers including species of the genera *Defluviitoga* and *Acetomicrobium* (Supplementary Table S2).

At genus level, *Syntrophomonas* spp. (Clostridiales) revealed a significantly higher abundance when unsterile soil as inoculum (Supplementary Figures S2A,B) and correlated positively with CH_4_ production (rs = 0.76, *p* < 0.001). Besides, *Herbinix* spp., vadinBB60 group, *Cryptanaerobacter* spp., and *Hydrogenispora* spp. showed higher relative abundances in the samples when unsterile soil (Su DFSs) was used, while DTU014_genus was higher in the samples when sterile soil (Ss DFSu) was used (Supplementary Figure S2B).

Regarding the archaeal community structure, Methanosarcinales were dominant in the samples using AG as inoculum, while Methanomicrobiales were dominant in the AP-inoculated samples, respectively (Figure 4). In contrast, acetoclastic and hydrogenotrophic methanogens were more or less equally represented in the samples using sterile soil as inoculum (Ss DFSu). Comparing the relative abundances in the control samples using sterile or unsterile soil revealed a significantly higher abundance of both Methanomicrobiales and Methanosarcinales in the samples containing unsterile soil as inoculum (Figure 4 and Supplementary Figure S2), which was reflected in a higher cumulative CH_4_ production (Figure 3A). Methanomicrobiales (*Methanoculleus* sp.) and Methanosarcinales (*Methanosarcina* sp.) were further identified as the most significant biomarkers using LEfSe (Supplementary Table S2).

With this information we started an attempt to identify key players that relate to stable and improved CH_4_ production during anaerobic digestion in the samples with soil-derived inocula. In general, methanogens were omnipresent throughout all approaches, however, as many of them were below detection limit due to their low abundance before incubation in the initial soil, it was rather difficult to draw concrete conclusions about their origin or relate them to specific conditions. In a few cases, however, we succeeded in tracing the origin and assigning specific OTUs to the soil-inoculated samples. In this context, soil derived *Methanosarcina* spp. turned out to be dominant members of the anaerobically incubated microbial community and key player in the carbon flow toward methane (Supplementary Figure S3B). Nevertheless, it became quite clear that the significantly higher CH_4_ yields achieved in the soil-inoculated samples could not be attributed to the activity of single species but rather rely on the overall occurrence of methanogens (defined as the sum of all reads) that significantly exceeded the ones calculated for the other soil/DFS variations.

Putative functional profiling showed a high number of predicted genes encoding for enzymes involved in methane metabolism that specifically related to the samples combining Su and DFSs (Supplementary Figures S4A,B). In this context, the genes encoding the tetrahydromethanopterin *S*-methyltransferase- (MTR), methyl-coenzyme M reductase- (MCR), and heterodisulfide reductase (HDR) complex required for the terminal reactions of methane formation were found to be significantly higher and at least two to three times more abundant in the samples using unsterile soil as inoculum compared with those observed in the other samples (Supplementary Figure S4B). Regarding interrelated pathways toward CH_4_, predicted genes participating in the fermentation of butyrate to CH_4_ and CO_2_ and those associated with the methylmalonyl-CoA pathway of propionic acid fermentation were also found to be significantly increased in the samples using unsterile soil and sterile DFS (Supplementary Figure S4A).

Methane monooxygenase (MMO) was only found in higher abundance in the soil samples *per se*, while being completely absent in all enriched (anaerobic) approaches. In this context, MMO constitutes the key enzyme of CH_4_ oxidation (Supplementary Figure S4C) carried out by methanotrophic Bacteria that are ubiquitously present in soils. Nevertheless, it has to be kept in mind that functional predictions were derived from taxonomic data and should thus be cautiously interpreted.

### Part II: Counteracting Effects of Soil-Derived Inocula During Stress Exposure

**FIGURE 5:**
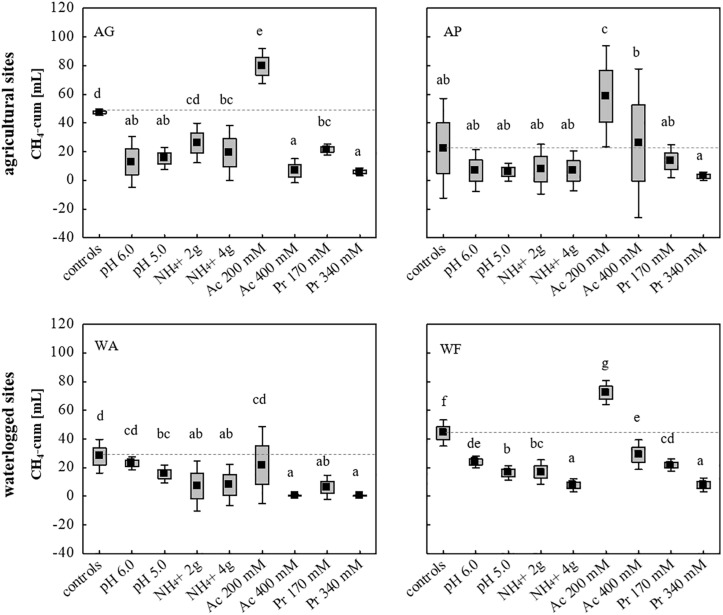
Cumulative CH_4_ production [mL] in 14 days of anaerobic incubation using two agricultural (AG, AP) and two waterlogged soils (WA, WF) as inoculum for anaerobic digestion. Boxes: mean ± SE, Whisker: mean ± 0.95 confidence interval, *n* = 9. Letters indicate statistically significant differences (*p* < 0.05) compared with the controls (indicated also by dashed lines). Study sites: *AG*, agricultural grassland; *AP*, agricultural pastureland; *WA*, waterlogged alluvial soil; *WF*, waterlogged fen. Stress (low and high load): *Ac*, acetate; *Pr*, propionate.

Using all nine initially chosen soils as inocula, the tolerance of the microbial communities toward low and high concentrations of NH_4_^+^ (2 and 4 g L^–1^), acetate (200 and 400 mM), propionate (170 and 340 mM) and initial pH values of 5.0 and 6.0, was evaluated using unsterile soil as inoculum and sterile DFS as substrate (Su DFSs), with each approach being anaerobically incubated for 14 days (Figure 1). The cumulative CH_4_ production achieved in selected four (AG, AP, WA, WF) out of nine original soils is summarized in Figure 5 (the cumulative production achieved using the remaining five soils (AA, FF, FL, FP, WB) is not depicted as they all showed very low to negligible production efficiency under both undisturbed and disturbed conditions). Single parameter variation revealed that the addition of 200 mM acetate boosted methanogenesis and led to a significantly higher CH_4_ production during 14 days of anaerobic incubation compared with the samples without acetate (Figure 5). Due to the rather promoting than impeding effect, the addition of acetate was excluded from further experiments. The strongest decline in CH_4_ production was observed adding high doses (340 mM) of propionate resulting in a significantly lower CH_4_ production in all approaches compared with the controls (Figure 5). Although pH- and NH_4_^+^-stress led to a decrease in CH_4_ production compared with the controls using AG, WA, and WF as inocula, the concentration applied (low and high dosage) showed no significant impact on the cumulative quantity at the end of the incubation period (Figure 5). Interestingly, neither pH nor high or low dosages of NH_4_^+^ as well as propionate concentrations of 170 mM hampered CH_4_ production during 14 days of incubation using AP as inoculum (Figure 5).

To verify whether soil-derived inocula are indeed beneficial to counteract stress exposure during AD, another test series was conducted with the four previously chosen soils. This time only high concentrations of the respective stress factors were applied but with the modification that the DFS and soil slurries were again added either sterile or unsterile (Su DFSs, Su DFSu, Ss DFSu, Ss DFSs). The anaerobic incubation period lasted 28 days in total, the corresponding cumulative CH_4_ production is shown separately for each of the four soils in Supplementary Figures S5, S6. Regarding stress exposure during anaerobic digestion, the pH-stressed samples followed a similar trend as observed for the controls and achieved the highest CH_4_ production in the samples combining unsterile soil and sterile DFS, however, with comparatively lower quantities within 28 days (Supplementary Figures S5, S6). The cumulative CH_4_ production was highest using Su DFSs under pH-stress (all soils) and NH_4_^+^ stress (AG and AP), while WA and WF showed (although lower in quantity) a higher cumulative production combining Su DFSu (Supplementary Figures S5, S6). Sterile soil combined with unsterile DFS generally led to a higher cumulative CH_4_ production in the samples containing high propionate concentrations, although the obtained methane quantity was again significantly lower compared with the other additives (Supplementary Figures S5, S6). As was to be expected, combining sterile soil and sterile DFS again led to no CH_4_ production during the entire incubation period (Supplementary Figures S5, S6), which proved that the sterilization of soils and DFS was successful.

**FIGURE 6:**
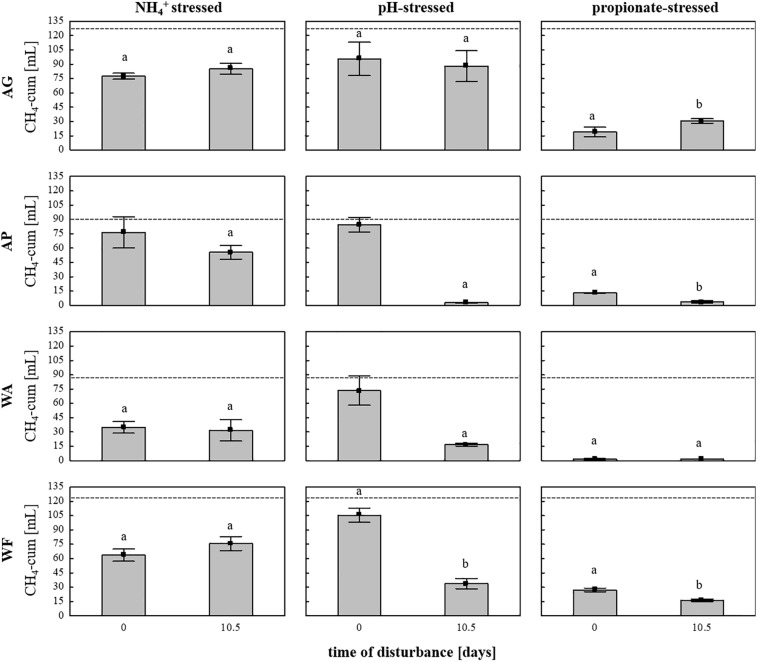
Cumulative CH_4_ production [mL] in 28 days of anaerobic incubation in the NH_4_^+^–, pH-, and propionate-stressed samples after a disturbance on day 0 (start-up) or after 10.5 days of incubation. Bars represent means (±SD), *n* = 3. Dashed lines indicate cumulative CH_4_ production in the controls (unstressed samples) after 28 days of anaerobic incubation Significant differences (*p* < 0.05) to the controls are indicated by characters, different characters indicate significant differences between disturbance times. *AG*, agricultural grassland; *AP*, agricultural pastureland; *WA*, waterlogged alluvial soil; *WF*, waterlogged fen.

Subsequently, the adaptation of the microbial communities toward parameters connected with common process failures was evaluated using the same four soils as inocula and sterile DFS as substrate, with stress (high load) being exposed on day 0 (start-up) or on day 10.5 to investigate possible different response traits during 28 days of anaerobic incubation. The cumulative CH_4_ quantities depending on soil inoculum, stress factor and exposure time are summarized in Figure 6. Even though all soils exhibit similar native pH values in the near neutral range (Supplementary Table S1), pH variation during start-up caused no significant differences in cumulative CH_4_ production compared with the controls, whereas varying the pH after 10.5 days of anaerobic incubation caused a significant decrease in cumulative CH_4_ production compared with the controls and the samples being stressed during start-up except for the samples using AG as inoculum (Figure 6). In contrast to pH variation, cumulative CH_4_ production during NH_4_^+^ inhibition turned out to be unaffected by time of stress exposure (Figure 6). Irrespective of the time of amendment (day 0 or day 10.5), high propionate concentrations again led to the strongest decline in CH_4_ quantities in all four tested soils, with very low production observed throughout the entire anaerobic incubation period, especially regarding WA and AP (Figure 6). In general, the production efficiency decreased as follows: pH > NH_4_^+^ > propionate when being stressed during start-up (day 0) and NH_4_^+^ > pH > propionate when being stressed after 10.5 days of incubation.

Evaluating the distinct structural differences in the microbial community associated with soil-derived inocula (Su DFSs) and disturbance revealed that the bacterial phyla were largely unaffected by varying the initial pH from 7.0 (controls) to 6.0. In contrast, in the samples exhibiting high propionate levels an increase in Clostridia from 35 to 48% (AG) and 37 to 43% (AP) could be observed (Figure 4), while being at a comparative level in the samples using unsterile DFS with either sterile or unsterile soil. Besides Clostridia (mainly *Caldicoprobacter* spp., M55-D21, and MBA03), *Defluviitoga* spp. (Petrotogales), *Petrimonas* spp. (Bacteroidales), and *Haloplasma* spp. (Haloplasmatales) as well as the genus SRB2 (Thermoanaerobacterales, Clostridia) probably associated with sulfate reduction (Twing et al., 2017; Takaki, 2018) became more abundant in the propionate-stressed samples compared with the controls. The latter were, however, characterized by significantly higher abundances of *Syntrophomonas* spp. and *Methanoculleus* spp. (Supplementary Figure S7) coinciding with the higher cumulative CH_4_ production.

In the context of NH_4_^+^ disturbance, Methanosarcinales remained dominant in the samples using AP as inoculum (Su DFSs) and replaced Methanomicrobiales in the samples using AG as inoculum as the prevalent order, which further coincided with biomarker discovery using LEfSe (Supplementary Table S2). In this context, the relative abundance of Methanosarcinales (mainly *Methanosarcina* spp.) rose from about 16 to 24% (AG) and from 24% to a climax of about 42% (AP) (Figure 4 and Supplementary Figure S7). The increase of Methanosarcinales was compensated by a substantial decrease in the relative abundance of Methanomicrobiales, particularly *Methanoculleus* spp. (AG: from 21 to 11%, AP: from 15 to 5%), compared with the samples without stress (Figure 4 and Supplementary Figure S7), which was also observed in the pH- and propionate-influenced samples. In contrast, no discernible trend was found regarding Methanosarcinales that remained (relatively) stable and seemed to play a key role in CH_4_ production in the samples with active soil-derived inocula even after being stressed (Figure 4 and Supplementary Figure S7). Contrarily, Methanomicrobiales outcompeted acetoclastic methanogens under propionate-stress, which was not the case in the samples using sterile DFS (Figure 4 and Supplementary Figure S7). Although accounting for minor parts of the total archaeal community, members of the order Methanomassiliicoccales were found in the samples combining Su DFSs with high propionate levels (Figure 4) and were identified as significant biomarkers when high concentrations of propionate were applied (Supplementary Table S2).

## Discussion

Soils harbor diverse and functionally dynamic microbial communities that are able to thrive under acidic as well as nitrogen-rich conditions and occupy natural niches in which various VFAs are major intermediates in the carbon flow to methane (e.g., wetlands, paddy- and rice fields) (Gan et al., 2012; Liu et al., 2017). This contradicts with the specified and (often) narrow range suitable for anaerobic digestion in biogas plants. Yet considering their natural tolerance toward parameters commonly associated with digestion failure, we investigated whether soil-borne (methanogenic) communities have the potential to improve CH_4_ production in biogas plants under (non)-disturbed conditions. Selection of suitable soil-derived inocula during *in situ* and incubation studies revealed that the waterlogged fen (WF) as well as the agricultural grass- and pastureland (AP and AG) showed positive CH_4_ fluxes and allowed satisfactory methane production (Figure 2). In this context, the positive *in situ* fluxes in the grassland (AG) and pastureland (AP) can most probably be attributed to inputs of methanogenic Archaea due to fermentation residue applications and grazing cattle excretions (as well as soil compaction and reduced aeration due to animal treading), while high water saturation creating ideal (anoxic) conditions allow the settlement of methanogenic Archaea in fens (Chasar et al., 2000; Blodau, 2002). qPCR conducted with the native soil samples of the four chosen soils provided further evidence for the abundance of methanogens (Figure 3B) that further coincided with cumulative CH_4_ production in these soils (Figures 3A,B).

Using four (out of nine) soils, we subsequently evaluated their suitability as inocula in AD processes and observed a consistently higher CH_4_ production in the samples using unsterile soil compared with the ones using unsterile DFS (Ss DFSu) (Figure 3A). Since autoclaving can promote substrate disintegration, we further tested this effect on both soil- and DFS-derived communities by adding unsterile DFS as inoculum in the same way as unsterile soil. Combining Su with DFSs led- as also seen in other incubations- to a significantly higher CH_4_ production compared with DFSu DFSs (Figure 3C), suggesting that autoclaving had a fundamentally mobilizing effect that is, however, more accessible by soil-derived than DFS-derived organisms, and thus probably due to differences in the microbial communities deriving from the inoculation sources. Particularly regarding the different output when soil is solely active suggests that the indigenous communities are able to process and convert the available nutrients better than the ones deriving from the DFS. As merely adding soil-derived communities fed with sterile DFS turned out to be more efficient in improving CH_4_ production than the original DFS community, might point to more diverse niches in soils than in AD systems. In this context, microbial diversity has been shown to play an important role in natural and engineered ecosystem function as it broadens the physiological spectrum and complementary response traits. As soon as these specific niches are occupied entirely by soil-derived communities without possible competitive disadvantages, they might be able to efficiently scavenge substrates from their environment even if available in low concentrations (Schneider, 2001; Albers et al., 2004; Wilkens, 2015) that, in turn, enhances CH_4_ production. Among Bacteria, the core bacterial phyla in the Su DFSs samples belonged to Firmicutes (foremost Clostridia) and Bacteroidetes (Figure 4). These two phyla have repeatedly been reported to be ubiquitous in nearly all microbial communities involved in CH_4_ production (Klocke et al., 2007; Sundberg et al., 2013; Solli et al., 2014), which was attributed to their high metabolic versatility and stability (Garcia et al., 2011; Kampmann et al., 2012). Both are prevalent at the initial stages of AD (Rivière et al., 2009; Buhlmann et al., 2019) and either involved in the metabolization of sugars and amino acids into the intermediary products acetate, H_2_, and CO_2_and thus provide substrates for acetogens and methanogens (Mara and Horan, 2003; Tracy et al., 2012) or in the degradation of VFA and thus prevention of methanogenesis inhibition (Krakat et al., 2011). This coincides with the significantly higher relative abundance of Firmicutes, particularly Clostridiales, in the samples using unsterile soil and sterile DFS (Supplementary Figure S3A). For the archaeal community, the significantly higher relative abundance of methanogens in the samples using soil as inoculum (Su DFSs) was not only congruent with a higher CH_4_ production (Figure 3A) but was further supported by gene functional annotation, where we found a high number of key enzyme-encoding genes associated with CH_4_ metabolism (*mtr*, *mcr*, and *hdr*) (Supplementary Figure S4B). Among Archaea, Methanosarcinales and Methanomicrobiales turned out to be the two most abundant orders and constituted the two most significant biomarkers in the Su DFSs samples (Supplementary Table S2). Even though Archaea represent only 0.5–3.8% of all prokaryotes in soils (Timonen and Bomberg, 2009), we were able to identify methanogens, i.e., *Methanosarcina* spp., that originated from soil and were assertive throughout subsequent anaerobic incubation in the samples using soil as inoculum (Supplementary Figure S3B). Previous studies reported *Methanosarcina* spp. as heavy duty methanogens being able to use either acetate or H_2_ and CO_2_ to produce CH_4_ (Lins et al., 2014; Lackner et al., 2018). Compared with other methanogens, *Methanosarcina* spp. have also proven to be quite robust toward various impairments including total ammonium concentrations up to 7000 mg L^–1^, pH-shocks of 0.8–1.0 units, and acetate concentrations up to 15,000 COD (chemical oxygen demand) mg L^–1^ (De Vrieze et al., 2012). In the context of microbial resource management, Bonk et al. (2018) revealed that communities with a higher proportion of *Methanosarcina* showed higher stabilities in AD processes, with the proportion being successfully increased by discontinuous feeding. These results, together with the significantly higher CH_4_ production and positive correlation of *Syntrophomonas* spp. with butyrate and CH_4_ (rs = 0.58 and rs = 0.76, *p* < 0.001) observed in the samples combining Su DFSs, indicate an improved synergistic performance along with enhanced substrate utilization that might give an early advantage regarding digestion performance. Regarding stress exposure, Methanosarcinales further played a key role in methane formation and prevailed under deteriorated conditions (Figure 4 and Supplementary Figure S7). Somewhat surprising, Methanosarcinales propagated in the NH_4_^+^ stressed samples (Supplementary Figure S7), with lower rates but CH_4_ still being produced (Supplementary Figures S5, S6). Species of the genus *Methanosarcina* were further identified as significant biomarkers in these samples (Supplementary Table S2). This result was rather unexpected as hydrogenotrophic methanogens– in syntrophic relation with syntrophic acetate oxidizing bacteria (SAOB)– are generally considered prevalent under NH4^+^ stress (Westerholm et al., 2012). However, the mechanisms through which ammonium affect the engaged microorganisms in AD are complex, thus it is quite controversially discussed whether hydrogenotrophic or acetoclastic methanogens are more susceptible (Yenigün and Demirel, 2013; Chen et al., 2016). High ammonium concentrations are known to adversely affect methanogenesis (Chen et al., 2008; Fotidis et al., 2013, 2014; Rajagopal et al., 2013), yet the microbial community was able to tolerate these enhanced concentrations and we speculated that this was, in case of DFS, due to an adaptation of the microbial communities to the prevailing high ammonium concentrations (NH_4_^+^-N: ∼ 3200 mg N kg^–1^) in the digester from which the inoculum was sourced (Illmer and Gstraunthaler, 2009). This could also explain the higher CH_4_ production achieved in the samples using unsterile DFS when being stressed with NH_4_^+^ (Supplementary Figures S5, S6) as well as no significant differences in CH_4_ production irrespective of whether the community was stressed during start-up (day 0) or in later stages of fermentation (day 10.5) (Figure 6). In this context, it has been reported that 4000 mg TAN L^–1^ can be tolerated by acclimated methanogens (Yenigün and Demirel, 2013; Massé et al., 2014), with the concentrations used in the present investigation lying at the top end of the tolerance range. Taking the prevailing conditions [temperature: 37°C, initial (day 0) and final pH (day 28) of the soil slurries and DFS combined: 7.2 and 7.5, respectively] into account, the resulting concentrations of free ammonia are in the range of about 80–160 mg L^–1^ (calculated after Anthonisen et al., 1976). While ammonium is relatively harmless until reaching high concentrations, ammonia has been shown to be inhibitory even at low concentrations, i.e., above 30 mg L^–1^ (Fricke et al., 2007). These two compounds are in equilibrium but can be displaced dependent on the prevailing temperature and pH, with a shift toward easily and free membrane permeable NH_3_ occurring as both parameters increase (Fricke et al., 2007; Schnürer and Jarvis, 2010). Consistent with our results, De Vrieze et al. (2012) reported that a stable process can be established based on interactions between SAOB and *Methanosarcina* spp., with the acetoclastic pathway being dominant at low organic loading rates, low NH_4_^+^ concentrations and mesophilic temperatures. In the present study it can be assumed that due to their outstandingly high relative abundance of >40% (Figure 4 and Supplementary Figure S7) acetoclastic methanogens outcompeted other acetate consuming microorganisms. Possible explanations include that *Methanosarcina* spp. cluster together and form aggregates at high ammonia levels that increase the volume to surface ratio and thus their tolerance toward ammonia. Nevertheless, the SAO pathway (in syntrophic interaction with hydrogenotrophic *Methanoculleus* spp.) might have still been possible as indicated by low but still present mesophilic SAOB such as *Tepidanaerobacter* spp. and *Syntrophaceticus* spp. (Supplementary Table S3) when using unsterile DFS with either sterile or unsterile soil. Consistent with previous studies from Westerholm et al. (2016), the most abundant orders among Firmicutes in our investigation turned out to be largely unaffected by ammonium disturbance. In contrast, Thermotogae increased (up to 11%) as NH_4_^+^ concentrations increased in the samples using sterile soil with unsterile DFS (Figure 4) that are, like Firmicutes, capable of forming syntrophic relationships with hydrogenotrophic methanogens (Zakrzewski et al., 2012), while being entirely absent in the samples using unsterile soil as inoculum. In the present study, however, Thermotogae were represented solely by Petrotogales as the main order and *Defluviitoga* as the predominant genus.

Adding soil-derived inocula further proved to be an effective strategy to counteract unfavorable pH conditions by allowing satisfactory CH_4_ production at stable conditions. Control and pH-stressed samples did not differ significantly regarding their microbial community composition in the DFS samples inoculated with soil, however, they showed distinct differences from those combining Su DFSu and Ss DFSu as seen in a marked decrease in relative abundance of Methanosarcinales, Methanomicrobiales, and Clostridiales (Supplementary Figure S7). Among Clostridia, the genus DTU014 could clearly be assigned to the DFS-originating community, thus coinciding with its higher relative abundance in the incubations using unsterile fermenter sludge (Supplementary Figure S7). In contrast, Clostridiales and Petrotogales increased under propionate stress (Figure 4).

To date, only seven mesophilic syntrophic propionate-oxidizing Bacteria (SPOB) have been isolated within Firmicutes (e.g., *Pelotomaculum*) as well as δ-Proteobacteria (e.g., *Syntrophobacter*) that commonly prevail during disturbance (Dyksma and Gallert, 2019). As propionate constitutes (next to acetate and butyrate) a major intermediate during anaerobic digestion, with its degradation into acetate and H_2_/CO_2_ (and then to CH_4_) accounting for about 6 to 35% in total methanogenesis, a consistent turnover requires a syntrophic interplay with hydrogenotrophic methanogens to keep the H_2_ partial pressure at a sufficiently low level (Glissmann and Conrad, 2000; Gerardi, 2003). Comparing the unstressed and propionate-stressed samples using soil-derived inocula revealed a decrease in the relative abundance of Methanomicrobiales, coinciding with the low CH_4_ production rates during the entire incubation period (Figure 4). In this context, propionate can be considered as bottleneck affecting downstream processes during AD as its oxidation is endergonic (ΔG°′ of +76 kJ per mol) under standard thermodynamic conditions (Gerardi, 2003). Thermodynamically speaking, propionate oxidation is even more unfeasible than the one of butyrate, lactate and ethanol as the reaction requires 5–6 times lower H_2_ partial pressures compared with butyrate (Ahring et al., 2003; Li et al., 2012). Under stable digestion conditions, the volatile fatty acid concentration is generally low, thus slow growing acetogens responsible for their further conversion are also represented to a low extent. The small acetogenic community thereby only adapts slowly to an abrupt increase in VFA levels along with a delay in available substrates. As previously mentioned, SPOB such as *Syntrophobacter* spp., *Pelotomaculum* spp. and *Desulfotomaculum* spp. were detected in our samples and showed an increase under elevated propionate levels, however, they were present only in relatively low abundance. This coincides with the hypothesis of a small and slowly adapting community and might suggest that CH_4_ production was not entirely inhibited but rather delayed.

To conclude, soil-derived inocula proved to be effective in enhancing digestion performance as evidenced by a significantly higher cumulative CH_4_ production during anaerobic incubation. The increased productivity was accompanied by significantly higher relative abundances of methanogens in the samples using soil as inoculum, particularly Methanosarcinales and Methanomicrobiales, compared with the samples combining unsterile DFS with either sterile or unsterile soil. However, Bacteria (foremost Clostridiales) engaged in the preceding stages played an equally important functional role. These results indicate that the differences in CH_4_ production as well as tolerance toward disturbance are due to differences in the initial microbial community composition deriving from the different inocula. Soil-derived communities further proved to be beneficial in stabilizing anaerobic digestion processes against unfavorable pH as well as ammonium-stress due to their high ability to maintain metabolic resilience. Hence, soil-borne methanogenic communities can be considered suitable inoculation sources that determine the initial operational potential of AD and harbor significant potential for further exploitations.

## Data Availability Statement

The datasets presented in this study can be found in online repositories. The names of the repository/repositories and accession number(s) can be found in the article/Supplementary Material.

## Author Contributions

PI designed the study and helped with data interpretation and by contributing ideas. NP helped with bioinformatics, data interpretation, and contributed to the manuscript. MM performed the sample processing, data analysis, data interpretation, and writing of the original draft. All authors approved and contributed to the elaboration of the final manuscript (review and editing).

## Conflict of Interest

The authors declare that the research was conducted in the absence of any commercial or financial relationships that could be construed as a potential conflict of interest.
