# Supplementary material for: Soil-Derived Inocula Enhance Methane Production and Counteract Common Process Failures During Anaerobic Digestion
Source: Front Microbiol. 2020 Oct 20;11:572759. doi: 10.3389/fmicb.2020.572759 (PMC7606279; doi:10.3389/fmicb.2020.572759)
Supplement: Supplementary file 1 [file Data_Sheet_1.pdf]

Supplementary Material

Title:

Soil-derived inocula enhance methane production and counteract common process failures during anaerobic digestion

Authors:

Mira Mutschlechner, Nadine Praeg, and Paul Ilmer

Affiliations:

Universität Innsbruck, Department of Microbiology, Technikerstrasse 25d, 6020 Innsbruck, Austria

Correspondence:

Mira.Mutschlechner@uibk.ac.at

Accession number:

All sequence data obtained in this study were submitted to the National Center for Biotechnology Information (NCBI) Sequence Read Archive (SRA) and are accessible from the NCBI repository under BioProject **PRJNA637206.**

Supplementary Table S1: Characterization of the study sites including physical and chemical soil properties. Results are given as mean (± SD), n=3 for single study sites and n=9 for mean values of agricultural, forest, and water-logged sites. Significant differences ($p < 0.05$) are indicated by different characters.

Site	Coordinates	Altitude [masl]	Tair [°C]	Tsoil [°C]	DM [g g^{-1}]	OM [g g^{-1} DM]	pH	EC [µS cm^{-1}]	Total C [%]	Total N [%]	NH_4^+-N [µg N g^{-1} DM]	TC [µg g^{-1} DM]	TN [µg g^{-1} DM]	NPOC [µg g^{-1} DM]
AA	47°16'03.5''N<br>11°20'25.5''E	607	15.9	9.1	0.83<br>(0.01)	0.13<br>(0.11)	6.83<br>(0.12)	124.33<br>(5.13)	5.36<br>(0.82)	0.28<br>(0.03)	5.68<br>(0.47)	53.59<br>(4.84)	3.10<br>(0.51)	29.21<br>(1.86)
AG	47°14'40.8''N<br>10°51'37.3''E	529	12.3	6.2	0.65<br>(0.01)	0.16<br>(0.01)	6.96<br>(0.02)	202.27<br>(20.90)	8.15<br>(0.44)	0.72<br>(0.09)	5.58<br>(0.29)	83.24<br>(2.02)	12.91<br>(1.23)	38.27<br>(4.30)
AP	47°16'01.1''N<br>11°16'05.3''E	616	20.3	16.1	0.73<br>(0.02)	0.14<br>(0.03)	6.94<br>(0.14)	148.33<br>(14.29)	9.90<br>(1.02)	0.63<br>(0.08)	5.06<br>(0.08)	74.77<br>(4.31)	11.77<br>(7.94)	28.39<br>(2.77)
FF	47°16'44.1''N<br>11°22'15.9''E	905	16.9	12.2	0.74<br>(0.01)	0.12<br>(0.02)	4.11<br>(0.13)	19.67<br>(1.15)	4.55<br>(1.95)	0.25<br>(0.05)	3.25<br>(0.57)	112.28<br>(18.37)	4.87<br>(0.78)	96.80<br>(22.79)
FL	47°18'36.9''N<br>11°11'33.0''E	1180	12.5	10.9	0.65<br>(0.01)	0.15<br>(0.01)	6.64<br>(0.19)	55.00<br>(11.27)	5.57<br>(0.45)	0.34<br>(0.09)	6.44<br>(0.70)	106.08<br>(9.09)	7.45<br>(1.42)	54.74<br>(6.36)
FP	47°14'23.0''N<br>11°13'22.6''E	812	13.1	8.8	0.63<br>(0.05)	0.19<br>(0.08)	3.87<br>(0.57)	17.00<br>(6.08)	7.09<br>(2.87)	0.44<br>(0.12)	3.93<br>(0.32)	125.76<br>(38.39)	7.10<br>(2.53)	94.89<br>(39.75)
WA	47°15'57.9''N<br>11°18'56.8''E	574	18.5	11.3	0.60<br>(0.01)	0.15<br>(0.01)	7.03<br>(0.02)	181.33<br>(10.26)	11.67<br>(0.67)	0.62<br>(0.02)	5.24<br>(0.19)	115.23<br>(3.50)	14.86<br>(0.87)	44.80<br>(1.88)
WB	47°14'30.3''N<br>11°24'52.7''E	854	21.4	13.6	0.39<br>(0.03)	0.34<br>(0.04)	5.87<br>(0.02)	219.47<br>(3.20)	16.73<br>(4.25)	1.28<br>(0.32)	5.07<br>(1.44)	136.56<br>(14.68)	14.79<br>(1.99)	70.95<br>(8.92)
WF	47°14'16.8''N<br>11°24'52.2''E	835	22.0	13.9	0.34<br>(0.02)	0.47<br>(0.02)	6.60<br>(0.06)	114.33<br>(14.36)	25.43<br>(3.65)	2.30<br>(0.31)	8.36<br>(0.90)	157.47<br>(26.52)	25.31<br>(3.10)	92.60<br>(14.73)
	Average of agricultural sites				0.74^{a}<br>(0.08)	0.14^{a}<br>(0.06)	6.91^{b}<br>(0.11)	158.31^{b}<br>(36.90)	7.80^{a}<br>(2.10)	0.54^{a}<br>(0.21)	5.44<br>(0.40)	70.53^{a}<br>(13.65)	9.26^{a}<br>(6.15)	31.96^{a}<br>(5.48)
	Average of forest sites				0.67^{a}<br>(0.06)	0.15^{a}<br>(0.05)	4.84^{a}<br>(1.39)	30.56^{a}<br>(19.46)	5.74^{a}<br>(2.07)	0.34^{a}<br>(0.11)	4.54<br>(1.53)	114.70^{b}<br>(23.44)	6.47^{a}<br>(1.93)	82.15^{b}<br>(30.96)
	Average of water-logged sites				0.44^{b}<br>(0.12)	0.32^{b}<br>(0.14)	6.50^{b}<br>(0.51)	171.71^{b}<br>(46.96)	17.94^{b}<br>(6.67)	1.40^{b}<br>(0.76)	6.22<br>(1.82)	136.42^{b}<br>(23.82)	18.32^{b}<br>(5.57)	69.45^{b}<br>(22.47)

AA, arable land; *AG*, grassland with fermentation residue; *AP*, pastureland; *FF*, beech forest; *FL*, larch forest; *FP*, spruce forest; *WA*, alluvial soil; *WB*, bog; *WF*, fen. *masl*, meter above sea level; *Tair*, air temperature; *Tsoil*, soil temperature; *DM*, dry matter; *OM*, organic matter; *EC*, electrical conductivity; *Tot C*, total organic carbon; *Tot N*, total organic nitrogen; NH_4^+-*N*, plant available ammonium; *TC*, total carbon; *TN*, total nitrogen; *NPOC*, non-purgeable organic carbon.

Supplementary Table S2: Summary of the prokaryotic biomarkers at order level (and if available at higher resolvable tax level) for the applied additives and variations and for both studied soils detected by LEfSe at an LDA log score > 3.7. Methanogens are listed in bold. Unclassified genera are not included.

SOIL	STRESS	VAR	***LEfSe* biomarkers**	SOIL	STRESS	VAR	***LEfSe* biomarkers**
**AG**	Controls	Su DFSs	**Methanomicrobiales**<br>**(*Methanoculleus* sp.)**<br>Armatimonadetes<br>Clostridiales<br>(*Syntrophomonas* sp., *Cryptanaerobacter* sp., vadinBB60_group genus)<br>Fibrobacterales	**AP**	Controls	Su DFSs	**Methanosarcinales**<br>**(*Methanosarcina* sp.)**<br>Clostridiales<br>(*Syntrophomonas* sp., *Hydrogenispora* sp., *Cryptanaerobacter* sp.)<br>Izimaplasmatales
		Su/Ss DFSu	Synergistales<br>(*Acetomicrobium* sp.)<br>Armatimonadetes			Su/Ss DFSu	Bacteroidales<br>(FTLpost3 genus)<br>Clostridiales<br>(*Hydrogenispora* sp.)
**AG**	NH_4^+	Su DFSs	**Methanosarcinales**<br>**(*Methanosarcina* sp.)**<br>Clostridiales<br>(*Syntrophomonas* sp., Clostridia_MBA03 genus)	**AP**	NH_4^+	Su DFSs	**Methanosarcinales**<br>**(*Methanosarcina* sp.)**<br>Clostridiales<br>(*Syntrophomonas* sp.)
		Su/Ss DFSu	**Methanosarcinales**<br>**(*Methanosarcina* sp.)**<br>Clostridia_MBA03 genus<br>Bacteroidales<br>(Rikenellaceae)<br>Pseudomonadales<br>Izimaplasmatales			Su/Ss DFSu	**Methanosarcinales**<br>**(*Methanosarcina* sp.)**<br>Clostridia_MBA03 genus<br>Pseudomonadales<br>Bacteroidales<br>(*Proteiniphilum* sp.)
**AG**	pH 6.0	Su DFSs	Clostridiales<br>(*Hydrogenispora* sp., *Pelotomaculum* sp.)<br>Sphingobacteriales<br>(*Lentimicrobia* sp.)<br>**Methanomicrobiales**<br>**(*Methanoculleus* sp.)**	**AP**	pH 6.0	Su DFSs	Bacteroidales<br>(*Macellibacteroides* sp.)<br>Clostridiales<br>(*Syntrophomonas* sp., *Pelotomaculum* sp., *Herbinix* sp., *Hydrogenispora* sp.)<br>**Methanomicrobiales**<br>**(*Methanoculleus* sp.)**
		Su/Ss DFSu	**Methanosarcinales**<br>**(*Methanosarcina* sp.)**<br>Bacteroidales<br>Clostridiales<br>(*Hydrogenispora* sp.)<br>Pirellulales<br>Izimaplasmatales<br>**Methanomicrobiales**<br>**(*Methanoculleus* sp.)**<br>Pla1_lineage			Su/Ss DFSu	**Methanosarcinales**<br>**(*Methanosarcina* sp.)**<br>Armatimonadetes<br>Bacteroidales<br>Synergistales<br>(*Acetomicrobium* sp.)<br>**Methanomicrobiales**<br>**(*Methanoculleus* sp.)**<br>Pirellulales
**AG**	propionate	Su DFSs	Clostridiales<br>(*Garciella* sp., vadinBB60_group genus)<br>Petrotogales<br>(*Defluviitoga* sp.)<br>**Methanomassiliicoccales**<br>**(*Methanomassiliicoccus sp.*)**<br>Thermoanaerobacterales<br>(SRB2 genus)	**AP**	propionate	Su DFSs	Clostridiales<br>(*Caldicoprobacter* sp.)<br>Bacteroidales<br>(*Petrimonas* sp.)<br>Thermoanaerobacterales<br>(SRB2 genus)<br>Bacillales<br>Petrotogales<br>(*Defluviitoga* sp.)<br>Clostridia_M55-D21genus<br>Haloplasmatales<br>(*Haloplasma* sp.)
		Su/Ss DFSu	Petrotogales<br>(*Defluviitoga* sp.)<br>Halanaerobiales<br>(*Halocella* sp.)<br>**Methanosarcinales**<br>**(*Methanosarcina* sp.)**<br>**Methanomicrobiales**<br>**(*Methanoculleus* sp.)**<br>Clostridiales<br>(Peptococcaceae)			Su/Ss DFSu	Petrotogales<br>(*Defluviitoga* sp.)<br>**Methanosarcinales**<br>**(*Methanosarcina* sp.)**<br>Clostridia_DTU014 genus<br>Halanaerobiales<br>(*Halocella* sp.)<br>Clostridiales<br>(Peptococcaceae)<br>Flavobacteriales<br>(*Flavobacterium* sp.)<br>Spirochaetales<br>(*Sphaerochaeta* sp.)

AG, agricultural grassland; *AP*, agricultural pastureland; *VAR*, Variation; *Su*, soil unsterile; *Ss*, soil sterile; *DFSu*, diluted fermenter sludge unsterile; *DFSs*, diluted fermenter sludge sterile. Biomarkers are sorted by descending LDA scores.

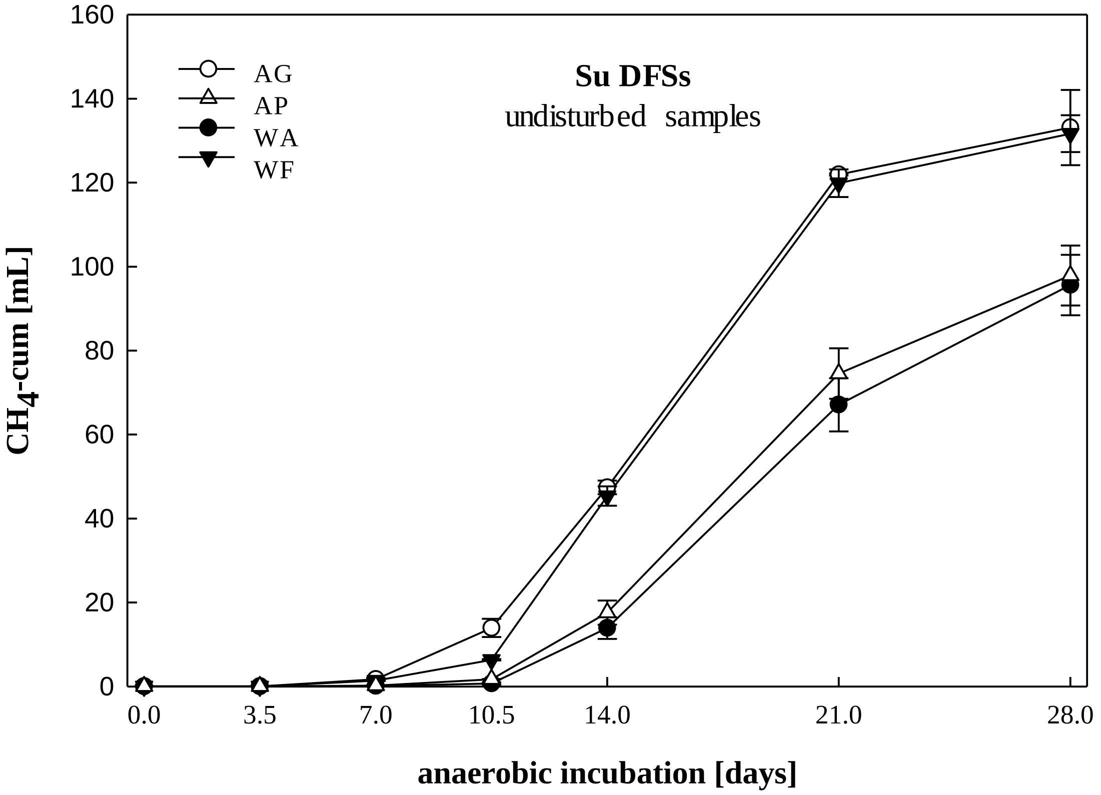

Supplementary Figure S1: Cumulative CH_4 production over time in the undisturbed samples using the two agricultural (AG and AP) and waterlogged soils (WA and WF) as inocula. Results represent means ± SD, n=3. *S*, soil; *DFS*, diluted fermenter sludge; *s*, sterile; *u*, unsterile. *AG*, agricultural grassland; *AP*, agricultural pastureland; *WA*, waterlogged alluvial soil; *WF*, waterlogged fen.

undisturbed samples (controls)

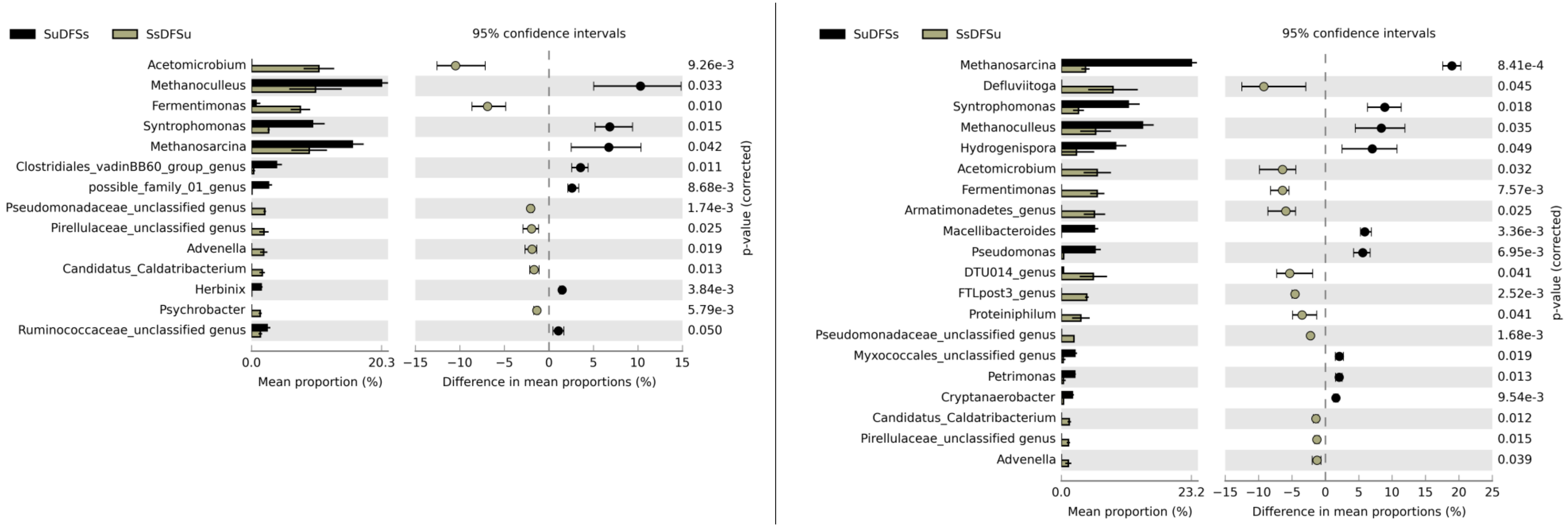

Supplementary Figure S2: Mean sequence proportions [%] of significant Bacteria and Archaea ($p < 0.05$, B-H-corrected, effect size > 1) of the undisturbed samples (controls) derived from AG **(A)** and AP **(B)** depending on soil/DFS variations. *Su DFSs*, soil unsterile and diluted fermenter sludge sterile; *Ss DFSu*, soil sterile and diluted fermenter sludge unsterile. *AG*, agricultural grassland; *AP*, agricultural pastureland.

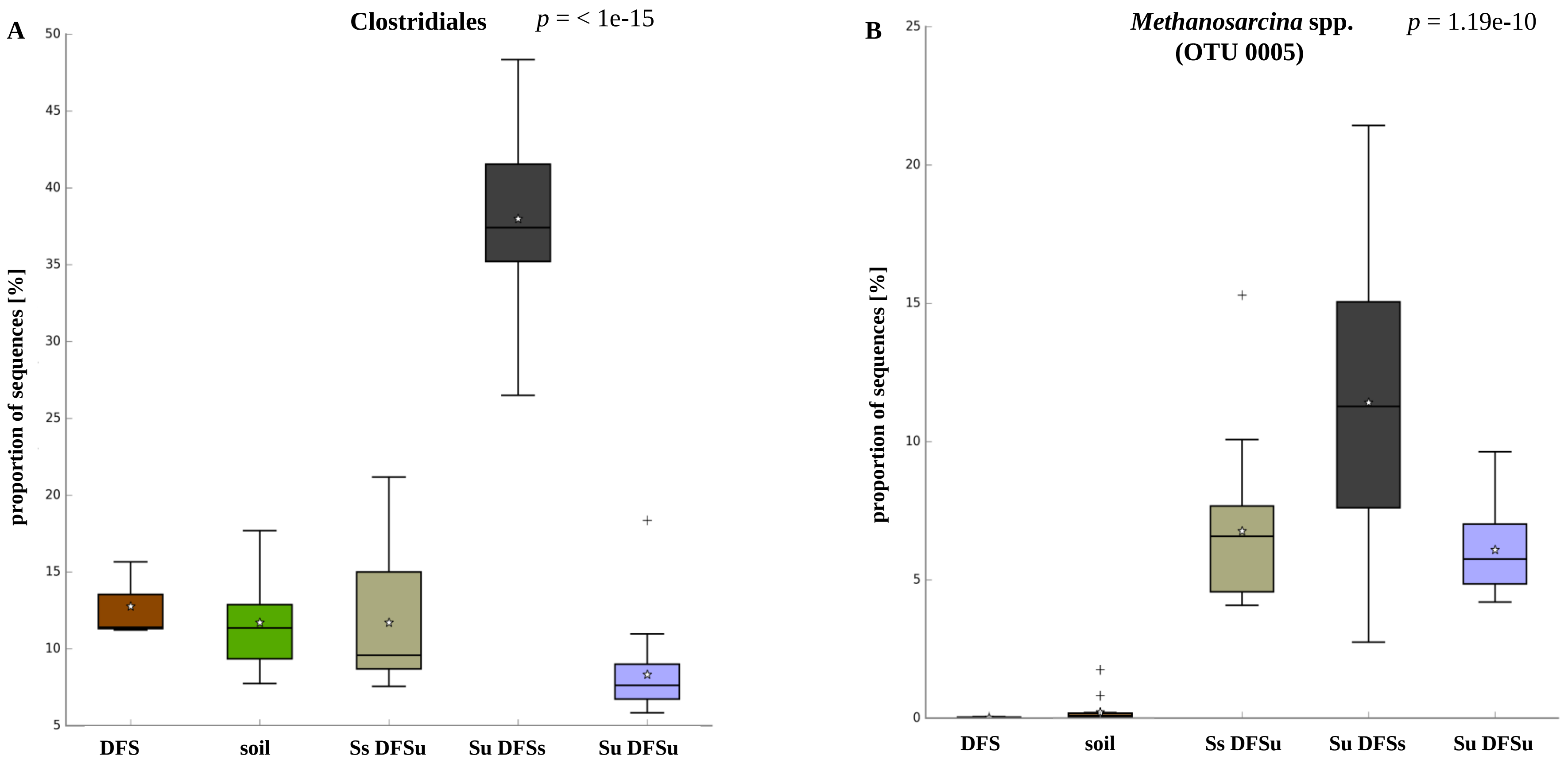

Supplementary Figure S3: Box-and-Whisker plots showing significant differences of (**A**) the order Clostridiales and (**B**) *Methanosarcina* spp. depending on the different inoculum variations.

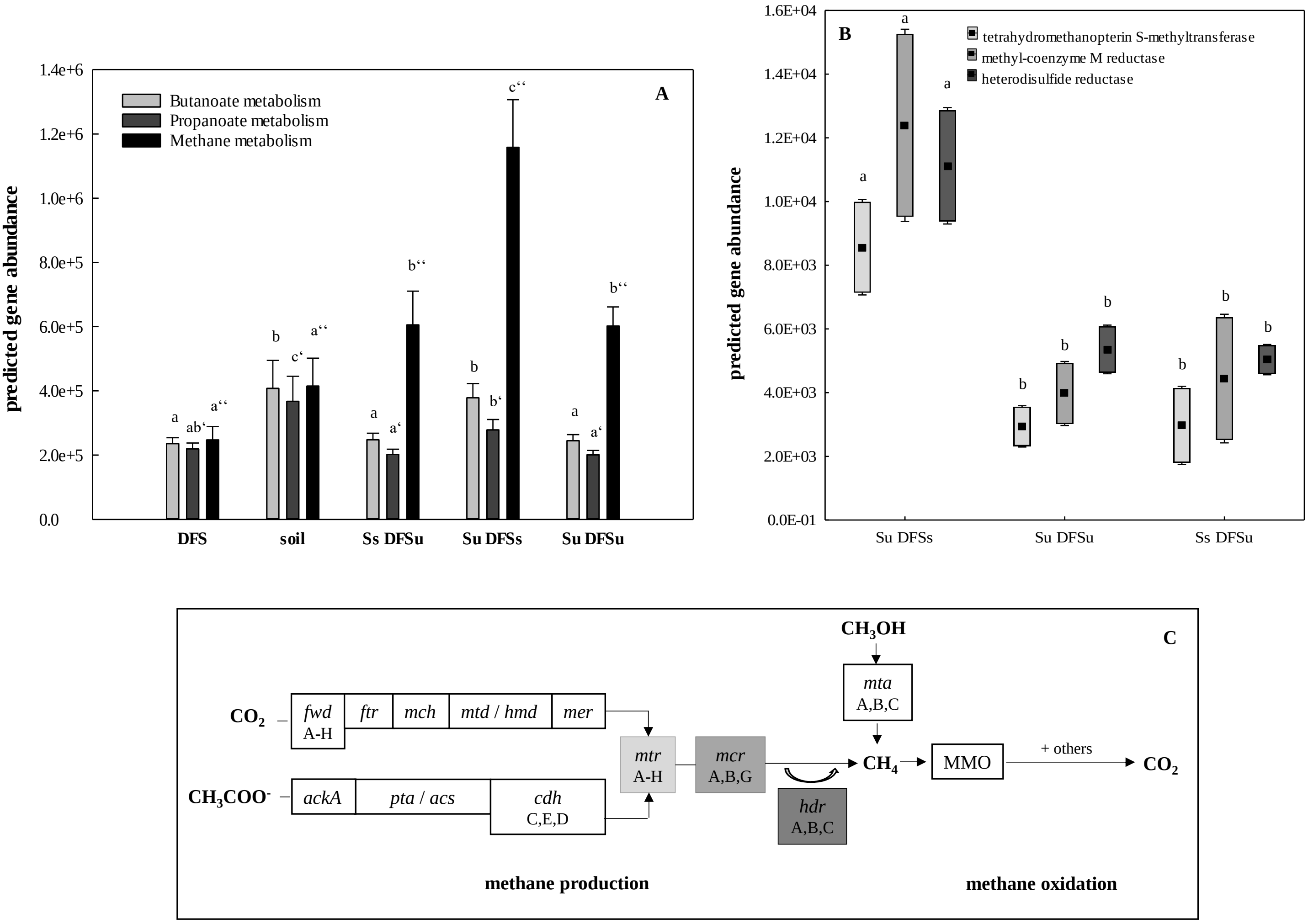

Supplementary Figure S4: (**A**) Function prediction of genes associated with butanoate-, propanoate- and methane metabolism. Bars represent means (± SD), n=3 (DFS) and n=6 (soil, variations), respectively. Significant differences ($p < 0.05$) due to soil/DFS variations are indicated by different characters. (**B**) Abundance of predicted genes responsible for CH_4 production. Boxes represent means (± SD), n=6. (**C**) Schematic overview of the specific enzymes involved in methane production and oxidation. Colors of boxes in (**C**) correspond to respective predicted gene encoding enzymes in (**B**). *CH_3OH*, methanol; *CH_3COO^-*, acetate; *Su*, soil unsterile; *Ss*, soil sterile; *DFSu*, diluted fermenter sludge unsterile; *DFSs*, diluted fermenter sludge sterile. *fwd*, formylmethanofuran dehydrogenase; *ftr*, formylmethanofuran–tetrahydromethanopterin N-formyltransferase; *mch*, methenyltetrahydro-methanopterin cyclohydrolase; *mtd*, methylenetetrahydromethanopterin dehydrogenase; *hmd*, 5,10-methenyltetrahydromethanopterin hydrogenase; *mer*, 5,10-methylenetetrahydromethanopterin reductase; *ackA*, acetate kinase; *pta*, phosphate acetyltransferase; *acs*, acetyl-CoA synthetase; *cdh*, acetyl-CoA decarbonylase/synthase complex; *mta*, methyl-Co(III) methanol-specific corrinoid protein; *mtr*, tetrahydromethanopterin S-methyltransferase; *mcr*, methyl-coenzyme M reductase; *hdr*, heterodisulfide reductase; MMO, methane monooxygenase (modified after Nolla-Ardevol et al., 2015).

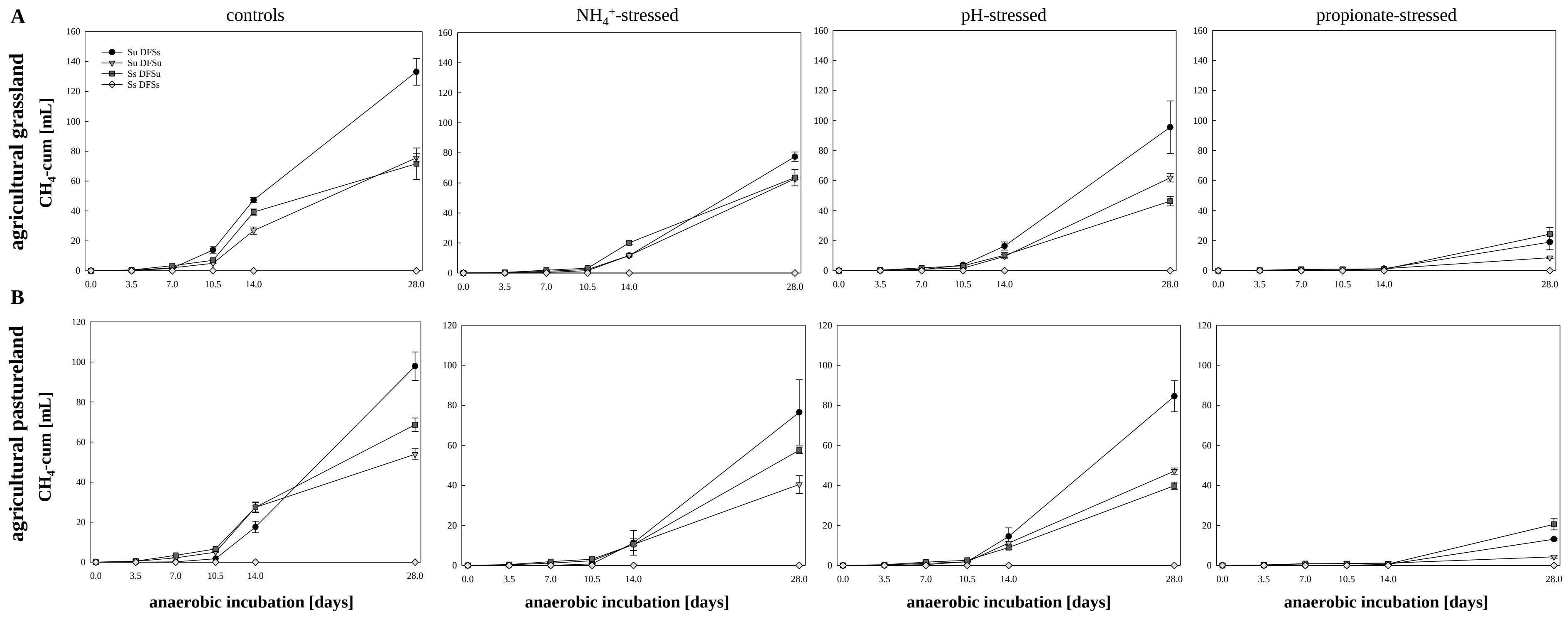

Supplementary Figure S5: Cumulative CH_4 production [mL] during 28 days of anaerobic incubation dependent on soil/DFS variation and stress exposure using AG (**A**) and AP (**B)** as soil-derived inocula. Results represent means (± SD), n=3. *S*, soil; *DFS*, diluted fermenter sludge; *s*, sterile; *u*, unsterile.

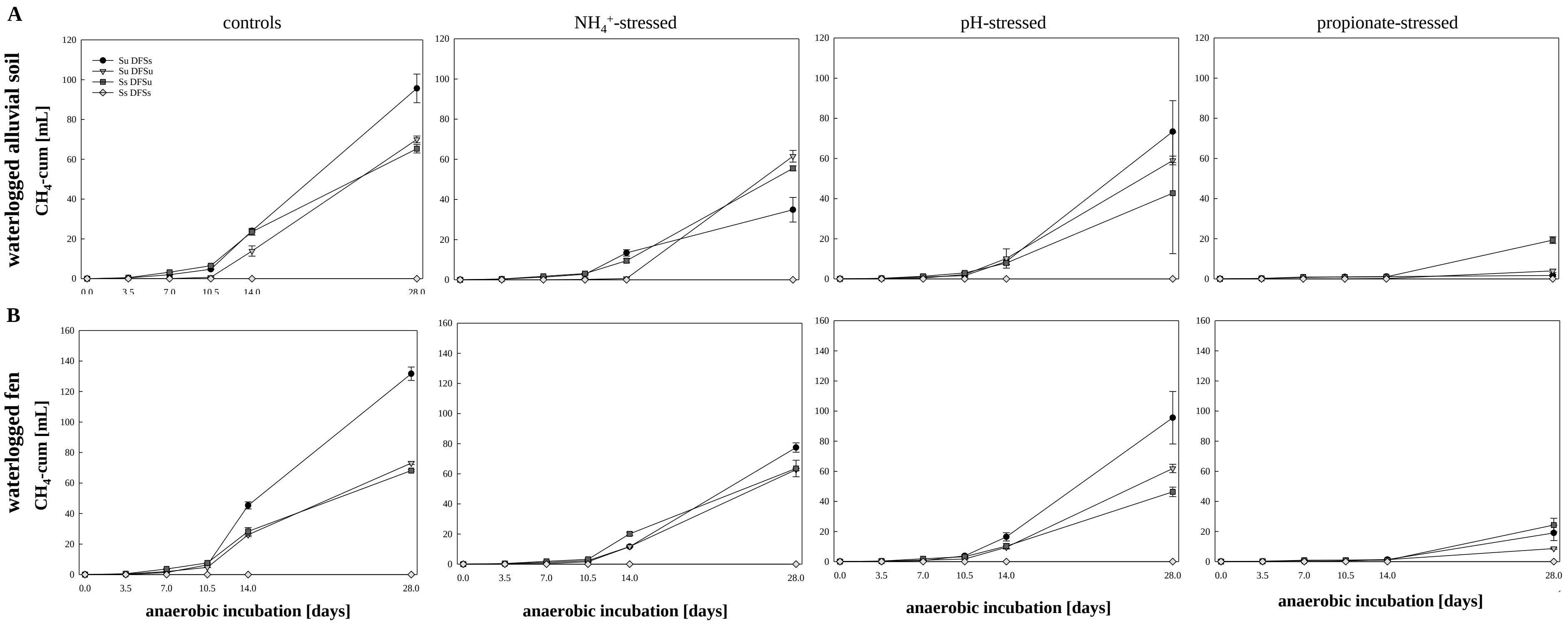

Supplementary Figure S6: Cumulative CH_4 production [mL] during 28 days of anaerobic incubation dependent on soil/DFS variation and stress exposure using WA (**A**) and WF (**B**) as soil-derived inocula. Results represent means (± SD), n=3. *S*, soil; *DFS*, diluted fermenter sludge; *s*, sterile; *u*, unsterile.

AG-inoculated samples **AP-inoculated samples**

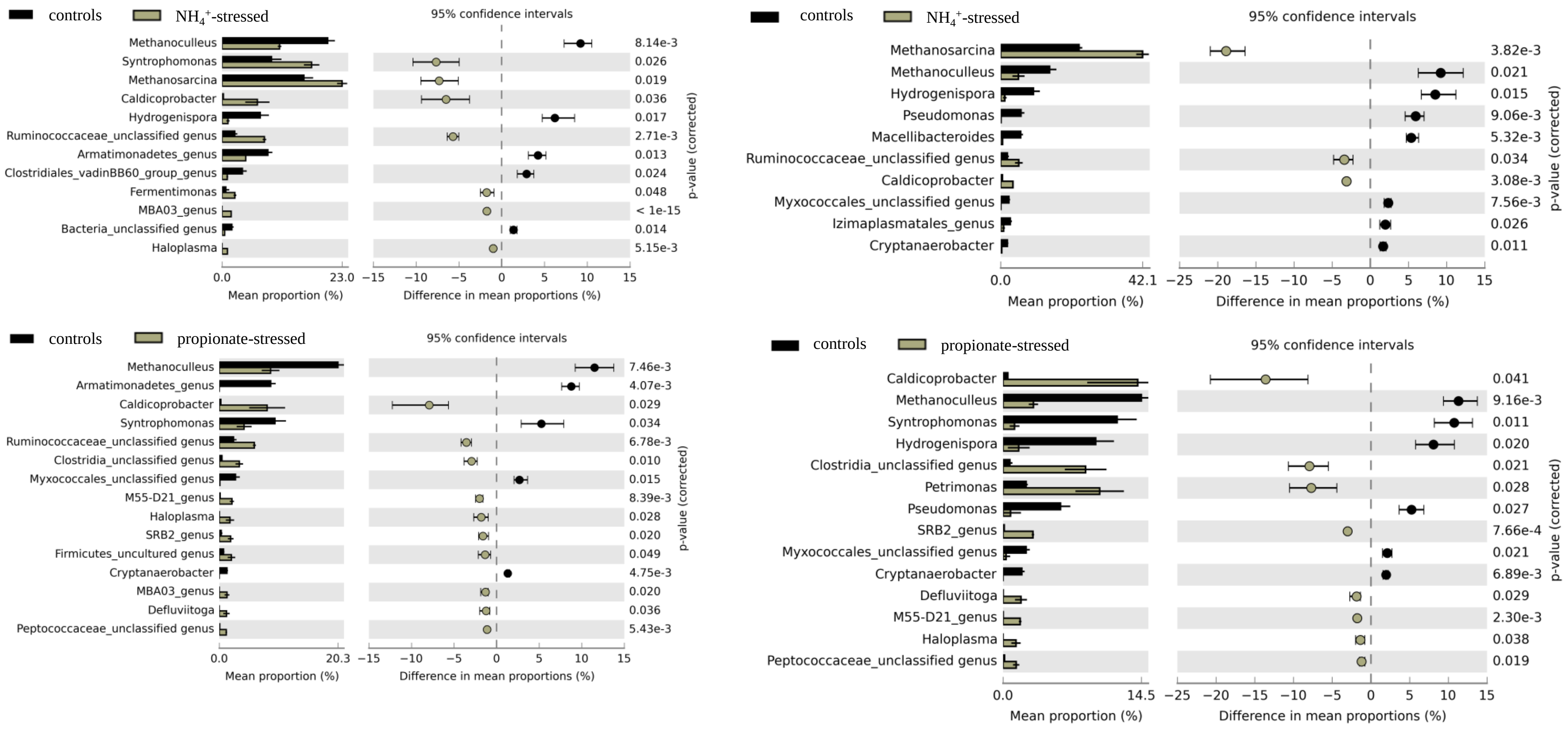

Supplementary Figure S7: Mean sequence proportions [%] of significant Bacteria and Archaea ($p < 0.05$, B-H adjusted, effect size > 1) of the NH_4^+- stressed (**A**, **B**) as well as propionate-stressed samples (**C**, **D**) inoculated with unsterile soil from AG (**A** and **C**) and AP (**B** and **D**) in comparison with unstressed samples (controls). *AG*, agricultural grassland; *AP*, agricultural pastureland.
